# Multimodal molecular mechanisms of octanoic acid (OA) against recurrent mastitis causing pathogens

**DOI:** 10.1007/s00253-026-13727-y

**Published:** 2026-02-05

**Authors:** Kai-Chen Hsu, Sanjay Prasad Selvaraj, Ming-Feng You, Wen-Chun Lin, Tsai-Ming Lu, Kuo-Hua Lee, Chau-Hwa Chi, Jyh-Yih Chen

**Affiliations:** 1 Department of Veterinary Medicine, National Taiwan University, No. 1, Sec. 4, Roosevelt Road, Taipei, Taiwan; 2 Molecular and Biological Agricultural Science Program, Taiwan International Graduate Program, Academia Sinica, Taipei, 11529 Taiwan; 3 Graduate Institute of Biotechnology, National Chung Hsing University, Taichung, 402 Taiwan; 4 Marine Research Station, Institute of Cellular and Organismic Biology, Academia Sinica, 23-10 Dahuen Road, Jiaushi, Ilan, 262 Taiwan; 5 Northern Region Branch, MOA-TRI, No. 207-5, Bi-Tou-Mian, Wu-Hoo Village, Si-Hoo Township, Miaoli, Taiwan; 6 iEGG and Animal Biotechnology Center and the Rong Hsing Research Center for Translational Medicine, National Chung Hsing University, Taichung, 402 Taiwan; 7 Institute of Cellular and Organismic Biology, Academia Sinica, Nangang, 115201 Taiwan

**Keywords:** Octanoic acid, Multimodal function, Transcriptomics analysis, Anti-adhesion and internalization, Toxin neutralization, Density functional theory, Molecular modelling

## Abstract

**Abstract:**

Recurrent bovine mastitis is a global concern that causes substantial economic losses and is exacerbated by pathogen internalization into mammary epithelial cells, and the emergence of antimicrobial resistance. These challenges necessitate the development of alternative antimicrobial strategies with multimodal activity. In this study, the naturally occurring molecule octanoic acid (OA) was evaluated for its antimicrobial efficacy and multitargeted mode of action against mastitis-associated pathogens. OA exhibited rapid bactericidal activity within 1 h and significantly reduced bacterial pathogenicity by attenuating toxin activity and inhibiting pathogen adhesion and internalization into epithelial cells. Transcriptomic analysis of *Staphylococcus aureus* revealed extensive OA-induced transcriptional alterations across multiple functional categories, including virulence regulation, stress response, metabolism, DNA replication and repair, membrane-associated functions, and transport systems, suggesting a broad cellular response to OA exposure. OA treatment also upregulated endogenous antimicrobial peptide (AMP) gene expression in MAC-T cells and did not induce detectable resistance even after 30 serial passages. Membrane perturbation was supported by molecular dynamics simulations and validated experimentally using DiBAC assays. In vivo toxicity assessment using *Galleria mellonella* demonstrated no observable toxicity up to 1000 mM OA. In addition, quantum chemical, physicochemical, and ADME/Tox analyses provided predictive insights into the chemical stability, drug-likeness, and safety profile of OA. Collectively, these findings suggest that OA exerts a multifaceted antimicrobial effect and represents a promising candidate for the development of next-generation antimicrobials targeting recurrent and resistant infections.

**Key points:**

• *Octanoic acid (OA) rapidly kills mastitis pathogens via multimodal mechanisms*.

• *OA prevents adhesion and internalization and mitigates toxicity in vitro and in silico*.

• *OA alters mRNA expression profiles, revealing key antimicrobial pathways*.

**Graphical Abstract:**

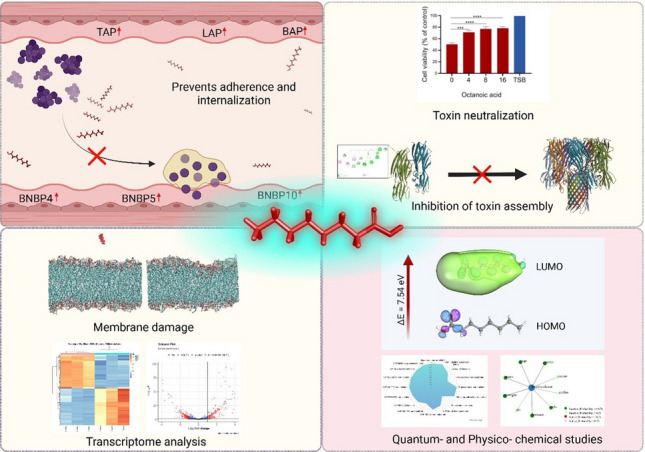

**Supplementary Information:**

The online version contains supplementary material available at 10.1007/s00253-026-13727-y.

## Introduction

 Bovine mastitis is a prevalent inflammatory condition caused by infection, which negatively impacts global milk production. This disease not only lowers milk yield and quality but also reduces profitability due to treatment costs; meanwhile, discarded milk from infected animals carries concerns over antibiotic residues (Morales-Ubaldo et al. 2023). Approximately 15% of dairy cattle worldwide are affected by mastitis, leading to an estimated annual economic loss of €2 billion in the European Union and similar losses in the USA (J. Wang et al. 2020). In China, the average incidence rate of bovine mastitis is around 33%, causing economic losses between ¥15 and ¥45 billion annually and severely hindering the development of the dairy industry (X. Wang et al. 2019).

Some of the most common mastitis-causing pathogens include *Staphylococcus aureus*, *Streptococcus agalactiae*, *Streptococcus uberis*, *Escherichia coli*, and *Enterococcus faecium*. Among these microbes, *S. aureus* is a predominant mastitis cause in many regions, with reported prevalence rates of 74% in Ethiopia and Canada, 47.2% in Italian herds, and 41% in France (Okmen et al. 2023). Evidence shows that *S. aureus* can invade mammary gland epithelial cells, forming intracellular reservoirs that disrupt the epithelial barrier, cause severe tissue damage, and trigger recurrent infections (Peyrusson et al. 2020). The severity of mastitis is influenced by the expression of bacterial virulence factors. A recent study in China revealed that over 70% of 219 *S. aureus* strains isolated from mastitis cases (in Beijing, Shandong, Shanxi, Inner Mongolia, Xinjiang, and Zhejiang) exhibited resistance to multiple antibiotics (Haddad Kashani et al. 2018). Similar patterns of multidrug resistance (MDR) have been reported for *S. aureus* strains isolated from North and South America, Asia, and Malta (D. Wang et al. 2015). Thus, there is an urgent need to find effective antibiotic alternatives for the treatment of bovine mastitis caused by MDR pathogens.

Antimicrobials with multimodal functions may offer a promising solution to overcome antimicrobial resistance. These agents can simultaneously target multiple bacterial components, such as cell membranes and mRNA, while modulating the host immune response to create synergistic protective effects. This approach can enhance treatment efficacy and reduce the potential for drug resistance to develop, addressing a common issue with conventional antibiotics that target specific bacterial functions. Drugs with multifaceted mechanisms may be particularly beneficial against MDR pathogens, as the combined actions lower the evolutionary pressure driving resistance (Chiang et al. 2018; Riduan and Zhang 2021). For example, antimicrobial peptides (AMPs) like epinecidin-1, LL-37, and TP4 exhibit multimodal actions on bacterial membranes, intracellular organelles, and host immune responses to combat infections (Yeh et al. 2025). However, there are several common drawbacks of AMPs, such as instability in serum and ions, degradation by proteases, and toxicity to host cells, that can prevent their successful clinical/commercial usage, leaving a need for further development of potential therapeutics (Mehraj et al. 2024; Narayana et al. 2015; Hazam et al. 2024; Selvaraj and Chen 2023).

Octanoic acid (OA) is a naturally occurring saturated medium-chain fatty acid (MCFA) that is present in coconut oil, breast milk, and bovine milk. It is non-toxic to humans and has been designated as Generally Recognized As Safe (GRAS) (CFR 184.1025) for use as a food additive (Korany and Aboelhadid 2024). Studies have shown that OA possesses anti-inflammatory and other beneficial biological properties (Sheng et al. 2022; Xinsheng Zhang et al. 2019). In previous research, we investigated the antibacterial and anti-biofilm activities of OA against mastitis pathogens (Lin et al. 2023). However, its detailed molecular mechanisms of action in bacterial targets and host systems remain largely unclear. In the current study, we explored the multifaceted properties of OA using in vitro, transcriptomic, and in silico approaches. OA was found to inhibit the initial stages of infection, including bacterial adhesion and internalization, upregulate AMP-related genes in mammary cells, alter mRNA transcription levels, and disrupt bacterial cell membranes. Density functional theory (DFT) calculations and physicochemical analyses further validated the drug-like properties of OA.

## Materials and methods

### Bacterial strains and MAC-T cell culture

*S. aureus* ATCC 12600 was purchased from the American Type Culture Collection (ATCC). A total of 18 clinical isolates (including strains from recurrent cases) were collected from the raw milk of dairy cows with mastitis in a cattle farm. The isolated strains of *Streptococcus *spp., *Staphylococcus *spp., *Escherichia coli*, and *Enterococcus faecium* were numbered based on the udder and isolate order. Details of the strains are listed in Table S1. The isolates were identified by 16S rRNA gene sequencing. Clinical isolates of *Streptococcus* spp. were cultured in Tryptone Soy Agar (TSA, Difco BD) with 10% horse serum (HS, Gibco). *Staphylococcus *spp., *E. coli*, and *E. faecium* strains were cultured in TSA. All clinical isolates and *S. aureus* ATCC 12600 were cultured at 37 °C in a shaking incubator set at 200 rpm. The MAC-T cells were maintained by serial passage in Dulbecco’s modified Eagle’s medium (DMEM, Gibco) supplemented with 10% fetal bovine serum (FBS, Gibco) (DMEM-10) (26, 27). Cells were cultured at 37 °C in a humidified atmosphere of 5% CO_2_. The cells were split when cultures reached 90% confluence.

### Bacterial killing kinetics analysis

Fresh overnight cultures of *S. aureus* ATCC 12600, clinical isolates *S. uberis* 6-2, *S. aureus* 10-9 and 13-1, and *E. coli* 8-8 and 9-8 were prepared. The cultures were adjusted to concentrations of approximately 10^7^ CFU/mL in milk (Kuang Chuan, Taiwan) containing either 50 mM OA or antibiotics at 0.1 mg/mL. Four antibiotics commonly used to treat bovine mastitis were tested, including ampicillin, cloxacillin, cefuroxime, and tetracycline (Sigma-Aldrich). Bacterial CFU counts were assessed at 0, 0.5, 1, 2, 4, 8, and 24 h.

### MCP adherence and internalization assays

The adherence and internalization of *S. aureus* ATCC 12600, clinical isolate *S. aureus* 10-9, *E. coli* 8-8 and 9-8, *Streptococcus lutetiensis* 2–6, and *S. uberis* 6-2 to MAC-T cells were assessed according to previously reported protocols with minor modifications (28, 29). In brief, 2 × 10^4^ MAC-T cells were seeded in a 24-well plate and cultured for 72 h, until the number of cells per well was approximately 2 × 10^5^ per well. Overnight cultures of bacterial cells were then treated at a multiplicity of infection (MOI) of 5 (1 × 10^6^ cfu/well) in the wells containing 72-h-cultured MAC-T cells. Treatments were administered in serum and antibiotic-free DMEM media (DMEM-0) in the presence or absence of 4 mM OA. For the adherence assay, after 2 h of incubation, the cells were washed three times in PBS. The number of bacteria adhering to the MAC-T cells’ surface was determined by evaluating the CFUs per well. For the internalization assay, after 4 h of incubation, the cells were washed three times with PBS. Then, 20 µg/mL lysostaphin (Sigma) or 0.1 mg/mL gentamicin (Sigma) was added in DMEM-0 to kill extracellular bacteria. After an incubation of 30 min at 37 °C, the cells were washed three times with PBS. Lysostaphin was used for *S. aureus*-treated MAC-T cells. Gentamicin was used for *E. coli*-, *S. lutetiensis*-, and *S. uberis*-treated MAC-T cells. The numbers of bacteria internalized by the MAC-T cells were determined by measuring the CFUs per well. Data are presented as the percentage of adherence and internalization compared to untreated control samples (100%).

### Cell viability assay using Alamar Blue

MAC-T cells were seeded at a density of 2 × 10^4^ cells per well in a 96-well plate using DMEM supplemented with 10% fetal bovine serum (DMEM-10). After 24 h of incubation to allow cell attachment, cells were treated with octanoic acid at the indicated concentrations. Following 24 h of treatment, cell viability was assessed using the Alamar Blue assay according to the manufacturer’s instructions. Fluorescence was measured using a microplate reader, and viability was expressed as a percentage relative to the untreated control group.

### Cytotoxicity of MCP conditioned medium (CM) toward MAC-T cells

*S. aureus* ATCC12600 and 18 clinical isolates were cultured at 37 ℃ with 200 rpm shaking for 24 h, and the supernatant was collected by centrifugation at 10,000 × *g* for 10 min. After centrifugation, the supernatants were filtered through a 0.22-µm membrane and used as CM. MAC-T cells were seeded 2 × 10^4^ cells per well in a 24-well plate. After 72 h of culture, the wells were washed with PBS and treated with 50% CM. Uncultured medium TSB or TSB with 10% HS in DMEM-10 was used as vehicle control. After 24 h incubation, MAC-T cell viability was analyzed using Alamar Blue (Invitrogen) according to the manufacturer’s instructions. Viability was measured to assess the effects of OA on cytotoxicity caused by *S. aureus-*secreted factors. Clinical isolate 13-1 was cultured for 16 h, and the cell pellet was resuspended at a concentration of approximately 10^9^ CFU/mL in TSB containing 0, 4, 8, or 16 mM OA. After 24 h incubation, the CM was collected by centrifugation and passed through a 0.22-µm filter. MAC-T cells were treated with 20% CM in DMEM-10. After 24 h of incubation, the viability of MAC-T cells was assessed by Alamar Blue. The pH values of the CM were not equalized in order to reflect the native extracellular environment generated by each clinical isolate, including the combined effects of secreted toxins, metabolites, and any pH changes they naturally induce.

### Expression of AMP genes in MAC-T cells

MAC-T cells were initially seeded at a density of 1 × 10^5^ cells per well in 6-well cell culture plates. After 72 h of incubation, various concentrations of OA (0, 1, 2, and 4 mM) were administered for 24 h, representing the uninfected stage. To model an infection stage, cells were exposed to a suspension of *S. aureus* ATCC 12600 at a concentration of 4 × 10^6^ CFU *S. aureus* per well in DMEM-0. Following 3 h of incubation, the cells were thoroughly washed with PBS. Subsequently, 20 µg/mL of lysostaphin in DMEM-0 was added to kill extracellular bacteria, followed by 30 min of incubation at 37 °C. The cells were then washed three times with PBS. To test drug effects on the infected stage, cefuroxime was added at a final concentration of 0.1 mg/mL along with different concentrations of OA (0, 1, 2, and 4 mM) in DMEM-10. After 24 h of incubation, the MAC-T cells were harvested, and total RNA was isolated according to the manufacturer’s instructions for the TRIzol RNA extraction kit (Invitrogen). Reverse transcription of total RNA was performed with ReverTra Ace® qPCR RT Master Mix (Toyobo). The transcript levels encoding bovine *tap*, *lap*, *defb*, *bnbd4*, *bnbd5*, and *bnbd10* were determined using quantitative real-time PCR (qRT-PCR) with an ABI StepOnePlus Real-Time PCR Instrument (Applied Biosystems, Foster, CA, USA). Table S2 lists the primers used for qRT-PCR. The gene expression levels were calculated using the 2^−ΔCt^ method, with β-actin expression serving as the endogenous control. The expression level fold-change was calculated relative to the untreated control (uninfected and OA-free treatment).

### Resistance development assay

In this study, *S. aureus* ATCC 12600 and clinical isolates *S. aureus* 10-9 and 11-1 were utilized to investigate the development of drug resistance. The induction of resistance to OA, cefuroxime, or cefuroxime in MHB medium containing 4 mM OA was assessed using a microbroth dilution assay. *S. aureus* strains were serially passaged in the presence of the drug to induce resistance. Initially, the MIC test was conducted. Wells were treated with sub-MIC (1/2 MIC) drug. Then, the cells were centrifuged at 6000 × *g* for 10 min to remove dead cells and residual antimicrobial agents. The remaining cells were then adjusted to approximately 1 × 10^6^ CFU/mL for the subsequent generation MIC experiments. The cells were incubated for 24 h at 37 °C according to the microbroth dilution assay protocol. MICs were determined in triplicate for generations 9–30.

### *Galleria mellonella *survival assay

Uniformly sized *Galleria mellonella* larvae showing no signs of melanization were selected for the assay. Octanoic acid solutions (200 mM, 500 mM, 1 M, and 2 M) were prepared in sterile saline. Untreated larvae and saline-injected larvae were included as control groups (*n* = 10 per group). Each larva was intrahaemocoelically injected with 20 µL of the respective solution into the last left proleg using a sterile microsyringe. Following injection, the larvae were maintained at room temperature. Larval survival was assessed at 24, 48, 72, and 96 h post-injection. Death was determined by the absence of movement upon gentle stimulation. Survival was calculated as a percentage of the initial number of larvae in each group.

### Transcriptome analysis

RNA-seq analysis was performed on *S. aureus* 13-1 under untreated conditions and after treatment with 4 mM OA for 4 h. Bacterial cells were washed three times with PBS by centrifugation. Total RNA was extracted from both control and treated groups, with three biological replicates per group. RNA-seq was performed by Welgene (Welgene, Taiwan). All RNA samples were prepared following the official protocol provided by Illumina. The SureSelect XT HS2 mRNA library preparation kit (Agilent, USA) was utilized for library construction, with size selection performed using AMPure XP beads (Beckman Coulter, USA). Sequencing was conducted using Illumina’s sequencing-by-synthesis (SBS) technology (Illumina, USA). Sequencing data in FASTQ format were processed through Welgene Biotech’s pipeline, which employs Illumina’s bcl2fastq v2.20 for basecalling. Adaptor removal and sequence quality trimming were carried out using Trimmomatic v0.36 with a sliding-window strategy (Bolger et al. 2014). RNA alignment was performed using HISAT2 (v2.2.1) (Kim et al. 2019). Transcript expression levels were normalized by computing transcripts per million (TPM) values with StringTie (v2.1.4) (Pertea et al. 2015). The whole genome sequence of *Staphylococcus aureus* KUH140087 was used as the reference genome. Preprocessing and normalization of RNA-seq data were conducted using the DESeq2 package, which was also used to identify differentially expressed genes (DEGs). Genes were considered significant if the adjusted *p*-value was less than 0.05 and |log2 fold change|≥ 1. A volcano plot was created to visualize DEGs. Hierarchical clustering and heatmap analysis, performed using the pheatmap package, displayed expression patterns of significant DEGs across samples. Principal component analysis (PCA) was carried out using the PCAtools package to evaluate sample clustering and identify potential outliers. Functional annotation and pathway enrichment analyses were performed using the clusterProfiler package (Yu et al. 2012), incorporating Gene Ontology (GO) terms and KEGG pathways. Annotation data were obtained from DAVID and KEGG (via BlastKOALA), and all analyses were executed in R (version 4.3.3).

### Molecular docking studies

Molecular docking studies were performed on OA with adhesion proteins and toxins of *S. aureus* (Selvaraj et al. 2024; Yeh et al. 2025). Crystal structures of target proteins were retrieved from the RCSB Protein Data Bank (PDB) (Figure S1, S2). Structure of fibronectin binding protein was downloaded from Alphafold database (ID: AF-A0A0H2XKG3). Proteins were prepared in PyMol by removing unnecessary water molecules and heteroatoms, and the structures were saved as PDB files. The binding sites of the proteins were identified by CastP online server and used for grid box generation. Ligands and proteins were converted to pdbqt format using AutoDock tools. Docking studies were performed in PyRx software using the AutoDock Vina algorithm. Binding energies, 3D docking poses, and 2D interaction diagrams were analyzed with BIOVIA Discovery Studio Visualizer and LigPlot.

### Molecular dynamics studies

MD simulations were performed using GROMACS 2023.5 software as described in the previous study (Hasannejad-Asl et al. 2024; Selvaraj et al. 2025). To replicate the interaction environment, systems underwent temperature and pressure equilibration within a solvent-involved simulation box. The CHARMM membrane builder server was used to position the OA molecule 3.5 nm from the bilayer membrane of *S. aureus* (POPG:TOCL1 = 46:32). Systems were neutralized with Na^+^ and Cl^−^ ions. After primary energy minimization, NVT equilibration was performed at 310 K using the V-rescale thermostat with a time constant of 0.1 ps. Periodic boundary conditions (PBC) are applied in all three dimensions (xyz). Three consecutive 500 ps NVT equilibrations were performed to stabilize the temperature. Long-range electrostatics were calculated using the Particle Mesh Ewald (PME) method with a cutoff of 1.2 nm for both Coulomb and van der Waals interactions. Semi-isotropic pressure coupling with the Berendsen algorithm was applied, maintaining 1.0 bar pressure in the x–y plane and z-direction. The LINCS algorithm constrained all bonds, and neighbor lists were updated every five steps using the Verlet scheme.

### Membrane depolarization assay

*S. aureus* 13-1 (10⁷ CFU/mL) was treated with OA and incubated for 4 h. The cells were washed and treated with bis-(1,3-dibutylbarbituric acid)trimethine oxonol [DiBAC₄(3)] at a final concentration of 2 µg/mL (Selvaraj et al. 2025). Following incubation, fluorescence intensity was measured using an ELISA plate reader with excitation and emission wavelengths set at 480 and 520 nm, respectively.

### Computational chemistry study

Quantum chemical calculations were performed using ORCA 6.0.1 open-access software by utilizing the B3LYP function with the 6-311G + + (d,p) basis set (Ferreira da Silva et al. 2024). Utilizing Koopman’s theorem, HOMO energy was correlated with ionization energy (I), while LUMO energy was used to estimate electron affinity. Additional chemical descriptors, including chemical hardness (η), electronegativity (χ), softness (S), global electrophilicity index (ω), and chemical potential (µ), were derived from HOMO and LUMO energy using standard equations (Siahaan et al. 2021):

- ΔE = *E*_*LUMO*_* − E*_*HOMO*_*.*
- *I* = − HOMO.
- *A* = − LUMO.
- η = 1/2 (*E*_*LUMO*_* − E*_*HOMO*_).
- χ = − µ ≈ ½ (*I* + *A*) ≈ ½ (*E*_*LUMO*_* − E*_*HOMO*_).
- *S* = 1/η.
- ω = µ^2^/2η.
- µ = −*E*_*LUMO*_ + *E*_*HOMO*_*)*/2.

### Physicochemical properties

The SwissADME online server was used to calculate the physicochemical properties of OA, and the drug-likeness was calculated based on Lipinski’s rule of five. The adsorption, metabolism, excretion, and toxicity properties of OA were estimated using the ADMETsar server. The toxicity profile was obtained from ProTox 3.0 tools (Banerjee et al. 2024).

### Statistical analysis

Experiments were performed in triplicate, and the data presented are representative of three independent experiments. Statistical analyses were conducted using GraphPad Prism 8.0 software. Statistical significance was assessed using one-way ANOVA followed by Tukey’s multiple post hoc test. For MCP adherence and internalization assays, comparisons with the untreated control were made using Student’s *t*-test. Values of *p* < 0.05 were considered statistically significant.

## Results

### Killing kinetics of MCPs

**Fig. 1:**
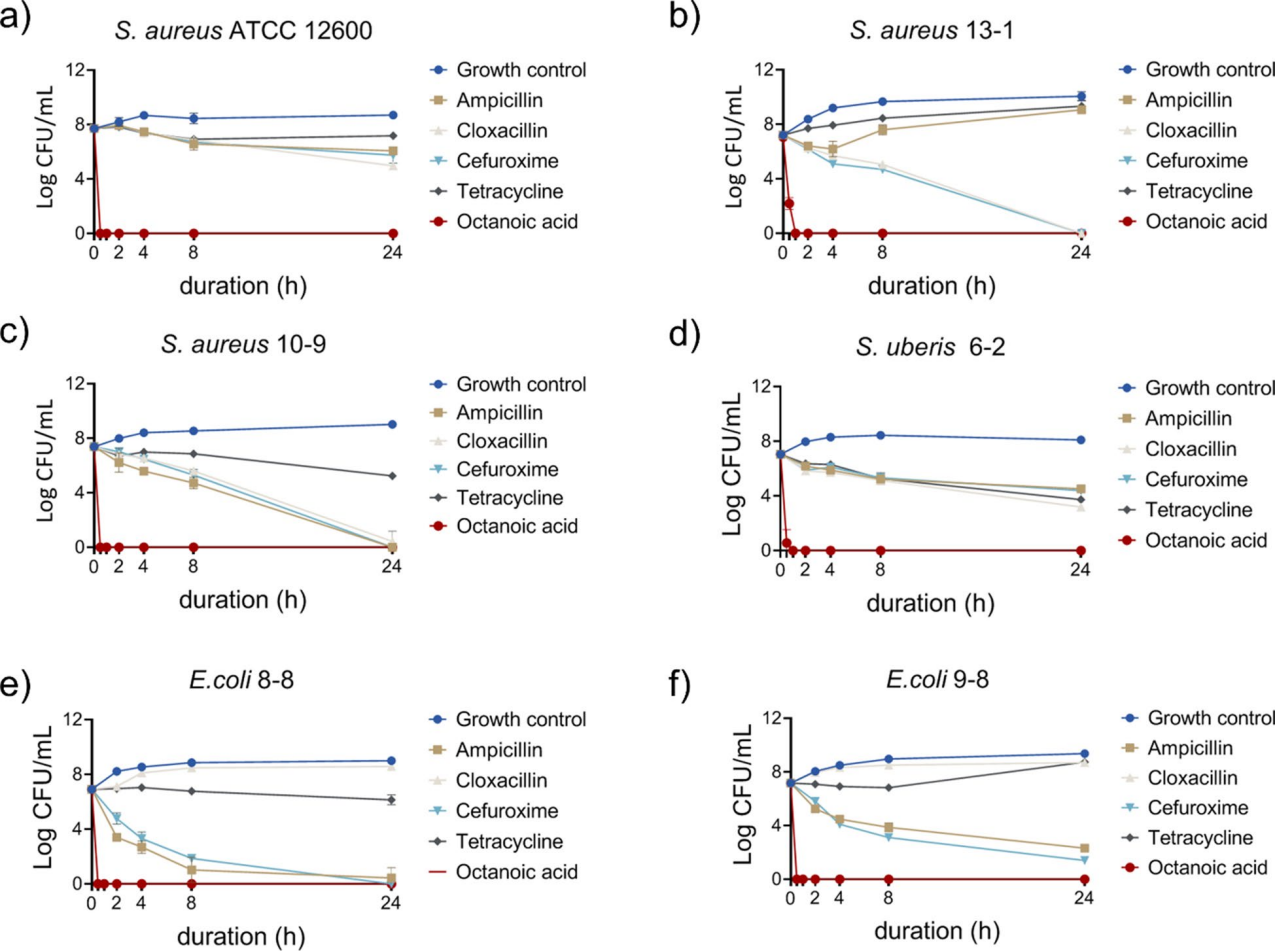
Octanoic acid (OA) clearance of mastitis-causing pathogens (MCPs). OA was applied against standard strain (Fig. 1**a**), clinical isolates (Fig. 1**b**–1**f**) and recurrent strains (Fig. 1**b**, 1**c**, 1**e**, 1**f**) at physiologically relevant millimolar concentrations, as required for medium-chain fatty acids. All values represent the mean ± SD of three individual replicates. OA treatment significantly reduced bacterial counts compared to growth controls (*p* < 0.05). The purpose of this figure is to demonstrate OA’s rapid bactericidal activity; direct comparison with conventional antibiotics, which act at nanomolar–micromolar concentrations, is not intended

Among the isolates, one strain of *S. equinus* (9-6), four strains of *S. aureus* (10-9, 11-1, 12-12, 13-1), two strains of *E. coli* (8-8, 9-8), and one strain of *E. faecium* (8-3) were found to be drug-resistant. The eradication kinetics of OA were tested against standard and clinical bacterial isolates, including strains from recurrent cases. Experiments were performed at a high bacterial load of approximately 10⁷ CFU/mL in milk, exceeding typical bacterial concentrations in infected cow udders. OA was applied at physiologically relevant millimolar concentrations (50 mM), as is required for medium-chain fatty acids to exhibit antimicrobial activity. Under these conditions, OA effectively reduced bacterial counts within 1 h. For reference, conventional antibiotics were tested at 0.1 mg/mL; although they also reduced bacterial counts, their mechanisms and potency differ fundamentally from OA, and direct potency comparisons should be interpreted with caution (Fig. 1). These results demonstrate that OA can rapidly inactivate mastitis-causing pathogens in milk and highlight its potential as an alternative or adjunctive treatment in intramammary therapy.

### Adherence and internalization in bovine mammary alveolar (MAC-T) cells

**Fig. 2:**
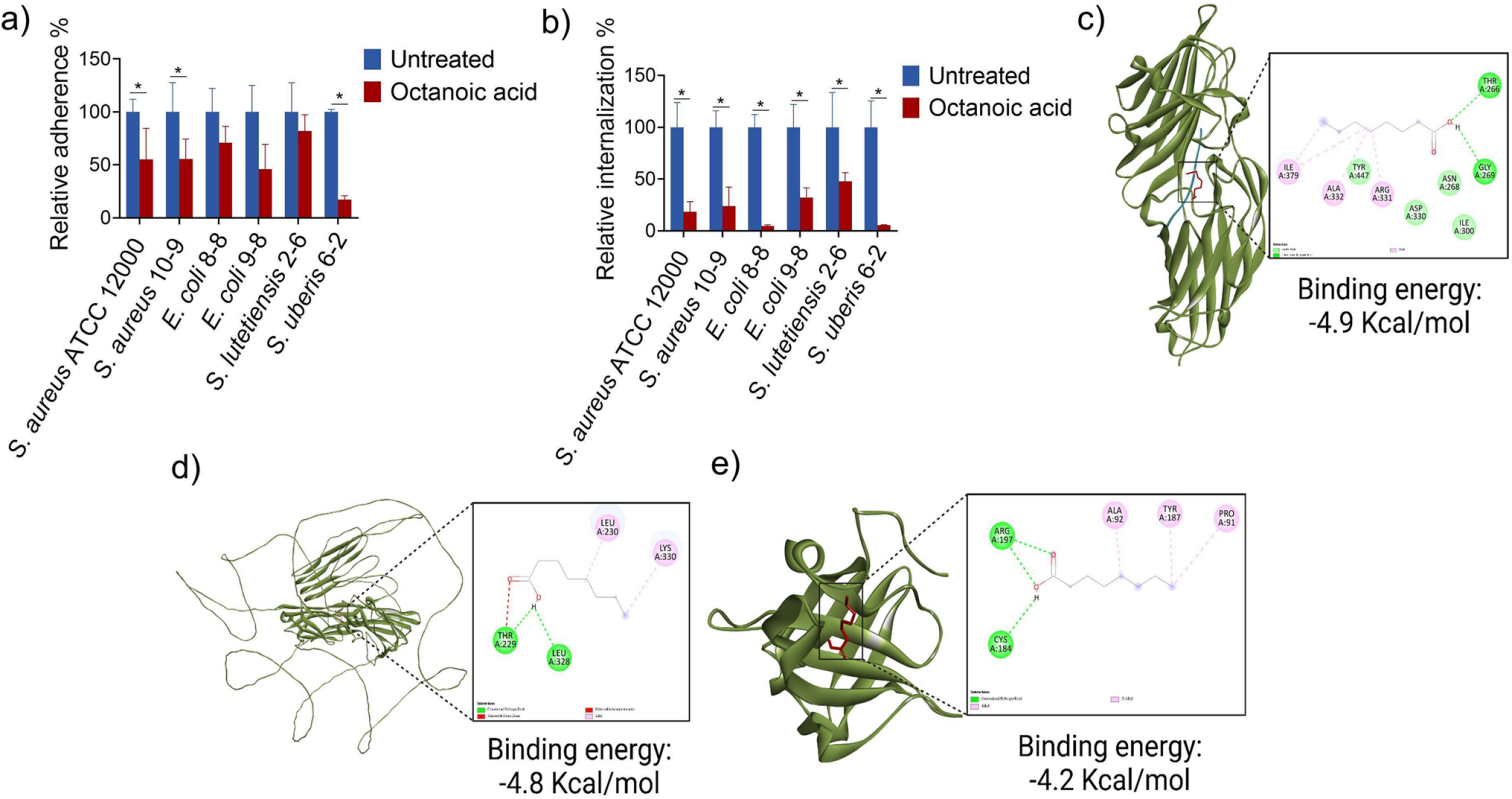
Adhesion and internalization assays and molecular docking analyses. **a** Adhesion test and **b** internalization assay. Molecular docking of OA with adhesion proteins, including **c** clumping factor B (PDB id: 4F20), **d** fibronectin binding protein (FnBP) (ID: AF-A0A0H2XKG3), and **e** sortase (PDB id: 1T2P). All values represent mean ± SD of three individual replicates. *p < 0.05 compared with the untreated control

Adherence of a pathogen to host tissues is considered a primary virulence mechanism, as this step facilitates colonization and internalization. While lethal doses of OA could efficiently eradicate MCPs in raw milk, we wanted to gather more comprehensive information about how pathogens and host cells react to low concentrations of OA. To understand the potential impacts of OA on internalization of MCPs by host cells, we measured pathogen adherence and internalization in MAC-T cells (Fig. 2a, b). The addition of 4 mM OA to the MAC-T cell culture medium significantly reduced the adhesion of *S. aureus* ATCC 12600, clinical isolate *S. aureus* 10-9, and *S. uberis* 6-2 to MAC-T cells (Fig. 2a). Furthermore, it decreased the internalization of several MCPs, including *S. aureus* ATCC 12600 and clinical isolates 10-9, 8-8, 9-8, and 6-2 (Fig. 2b). These findings suggest that OA can effectively reduce the invasion of pathogenic bacteria into host cells, potentially lowering the likelihood of mastitis relapse following treatment. The cell viability of MAC-T cells upon treatment with OA was performed using the Alamar Blue assay. OA at 4 mM concentration showed no significant cell toxicity compared to control wells (Fig S3). OA concentrations higher than 4 mM showed significant effects on MAC-T cells in vitro.

Molecular docking analysis was next performed to examine the potential of OA to bind adhesion proteins of *S. aureus*, such as clumping factor B (Fig. 2c), fibronectin binding protein (FnBP) (Fig. 2d), and sortase (Fig. 2e). The analyses showed significant binding energies with stable hydrogen bond formation for OA and each protein. These results suggest that the prevention of *S. aureus* adhesion and internalization might be due to the inhibition of the adhesion proteins, which could further inhibit colonization and lower virulence to mammalian cells and immune systems.

### Mitigation of cytotoxicity and in silicotoxin neutralization

**Fig. 3:**
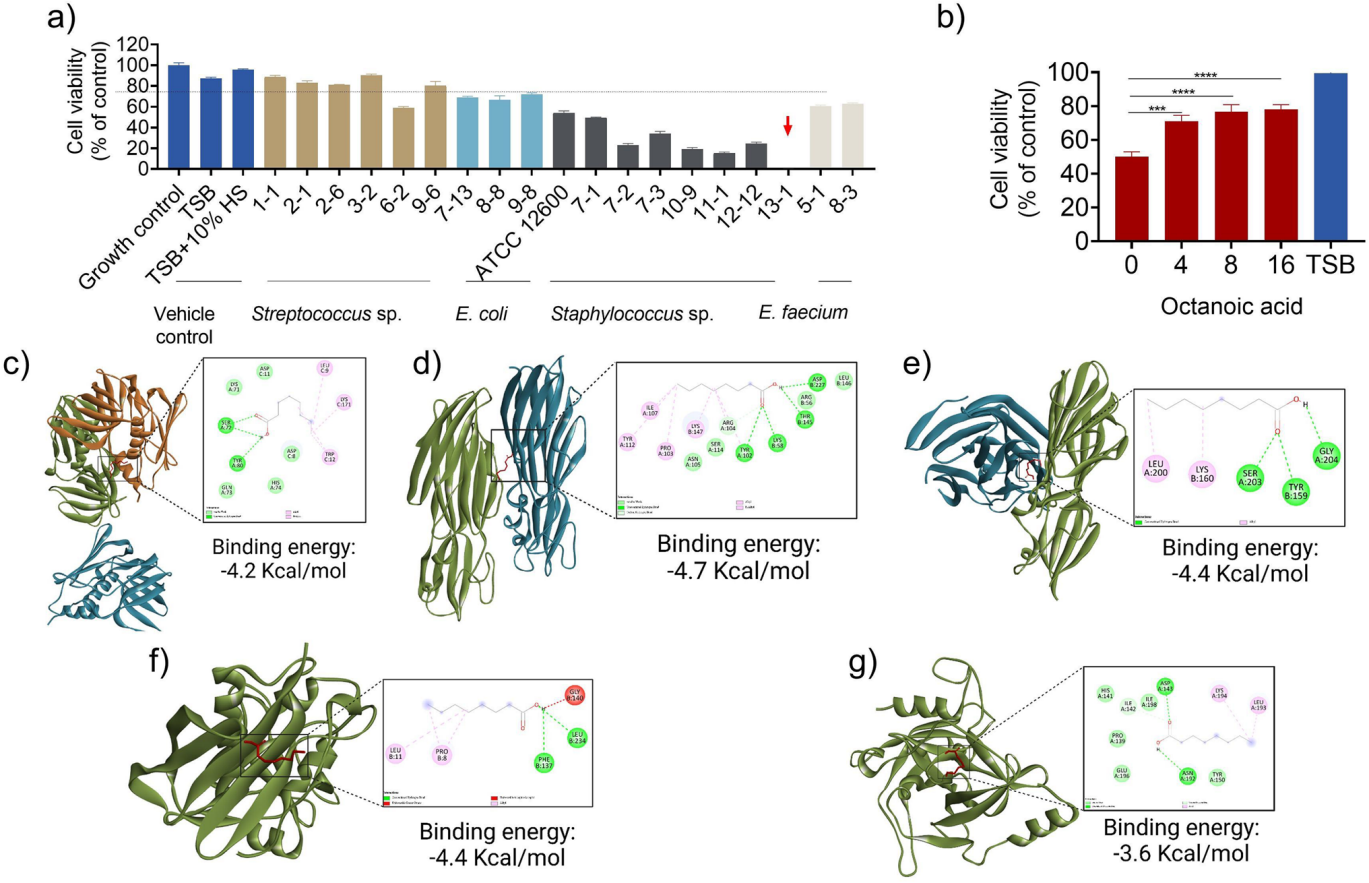
Toxin neutralization analysis. **a** Toxicity of conditioned medium (CM) from clinical isolates on MAC-T cells. **b** Cell viability after treatment with OA. Molecular docking analysis of OA with *Staphylococcal* toxins, including **c** α-hemolysin monomer (PDB ID: 6U3T), **d** γ-hemolysin monomer containing S and F subunits (PDB ID: 2QK7), **e** enterotoxin B (PDB ID: 1SBB), **f** toxic shock syndrome toxin (TSST) (PDB ID: 2QIL), and **g** exfoliative toxin B (PDB ID: 1QTF). Significant differences were determined by two-way ANOVA and Tukey’s multiple post hoc test (*p < 0.05; **p < 0.01; ***p < 0.001; ****p < 0.0001; ns, non-significant)

Antibiotic treatment of bovine mastitis is associated with a low cure rate, and residual pathogens remaining within mammary gland tissue after therapy can continue to secrete toxins. In severe cases, these toxins can cause the destruction of mammary gland tissue and reduce milk production. We therefore performed cytotoxicity testing on secreted factors from a total of 18 MCP clinical isolates, including *Streptococcus* spp*.*, *E. coli*, *Staphylococcus* spp*.*, and *E. faecium* (Fig. 3a). MAC-T cells were grown in 50% conditioned medium (CM) from each of the isolates. Cell viabilities in the presence of different *S. aureus* clinical isolates (i.e., 7-2, 7-3, 10-9, 11-1, 12-1, and 13-1) were less than 50% of control values. Notably, *S. aureus* clinical isolate 13-1 exhibited the highest level of toxicity among all tested strains. Since the secretome of *S. aureus* clinical isolate 13-1 caused severe cytotoxicity to MAC-T cells, it was further used to evaluate how OA affects the cytotoxicity of secreted factors. Since CM 50% showed complete cell death, CM 20% was utilized to create a measurable dynamic range that would allow us to observe changes in cytotoxicity. OA treatment (4, 8, and 16 mM) of *S. aureus *led to a significant increase in cell viability of MAC-T cells exposed to the secretome (20% CM) of treated cells, as compared to that of untreated *S. aureus* (Fig. 3b). These findings indicate that OA diminishes the toxicity of *S. aureus*-secreted factors, leading to better survival of mammary cells.

The mitigation of toxicity may be due to downregulation of toxin-associated genes or direct inhibition/neutralization of toxin molecules. α- and γ-hemolysins secreted by *S. aureus* tend to assemble as heptameric pores on the surface of the mammalian cells to promote the intracellular leakage and disrupt homeostasis. Significant binding of hemolysin inhibitors to monomeric proteins can inhibit the assembly of toxins, thus reducing the virulence of the pathogen. The data in Fig. 3c and 3d suggest that OA may predict to interact with α- and γ-hemolysin monomers. Similarly, OA exhibited potential to inhibit other *Staphylococcal* toxins, such as enterotoxin B (Fig. 3e), toxic shock syndrome toxin (TSST) (Fig. 3f), and exfoliative toxin B (Fig. 3g), which are associated with severe side effects on the host system.

### Upregulation of AMP genes in infected MAC-T cells

**Fig. 4:**
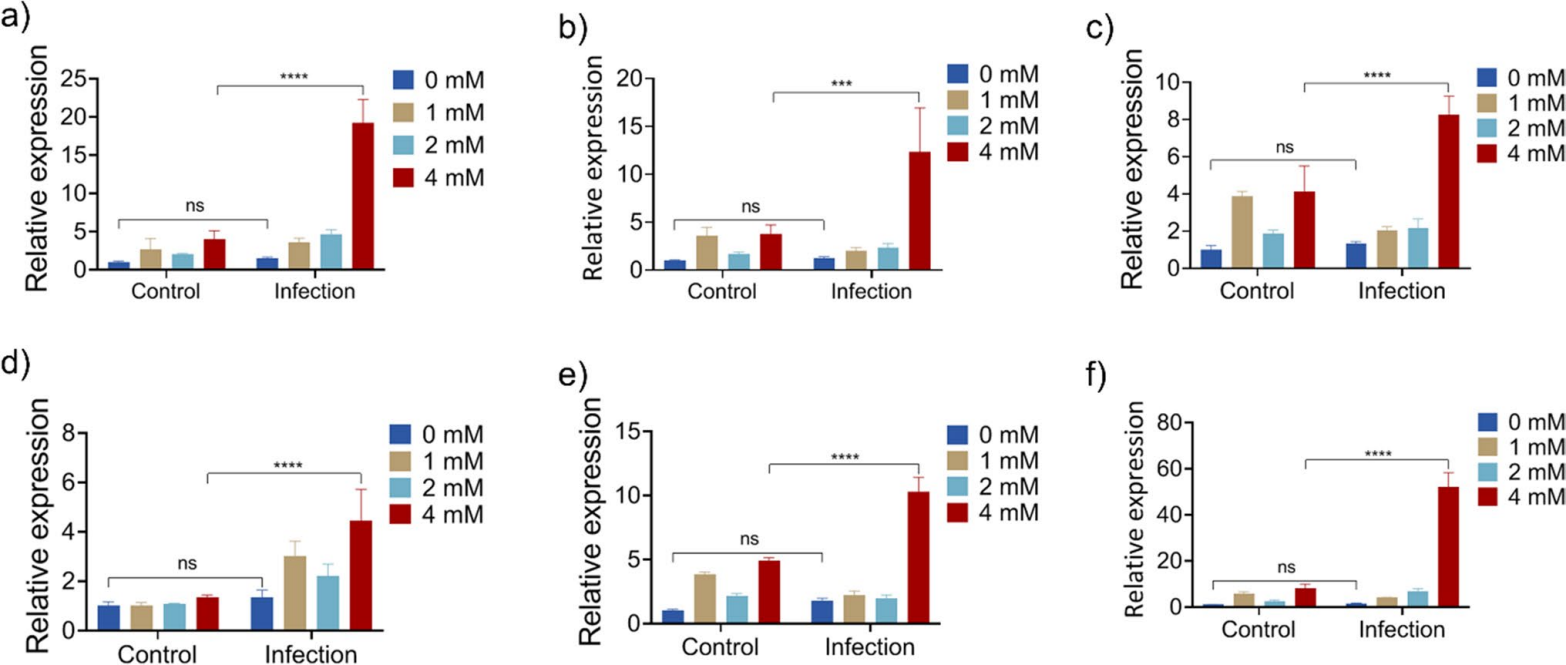
Regulation of endogenous AMP genes in MAC-T cells according to qPCR analysis*.* Relative expression levels are shown for **a** Tracheal Antimicrobial Peptide (TAP); **b** bovine tongue-derived lingual antimicrobial peptide (LAP), **c** epithelial cell-specific beta defensin (DEFB)1; **d** bovine neutrophil β-defensin-4 (BNBD-4); **e** bovine neutrophil β-defensin-5 (BNBD-5); **f** bovine neutrophil β-defensin-10 (BNBD-10). All values represent the mean ± SD of three individual replicates. Significant differences were identified by two-way ANOVA and Tukey’s multiple post hoc test (*p < 0.05; **p < 0.01; ***p < 0.001; ****p < 0.0001; ns, non-significant)

Modulation of host immunity along with direct bacterial killing is one of the vital mechanisms in multimodal functions. As key components of innate immunity, AMPs play a crucial role in combating infections. We therefore investigated the impacts of OA on AMP-related gene expression in MAC-T cells in both uninfected and infected conditions. As OA concentrations ranging from 1 to 4 mM showed no toxic effects on MAC-T cells, this concentration range was selected for gene expression analysis in the presence and absence of *S. aureus* ATCC 12600 (Fig. S3). After 24 h of infection, cell lysates were harvested, and quantification of the AMP gene expression was performed. There were no significant differences in the expression of AMP genes when comparing between uninfected and infected MAC-T cells without OA exposure. In the uninfected MAC-T cells, there were also no significant differences in the expression of AMP genes after OA treatment. It is noteworthy that in the presence of 4 mM octanoic acid, the expression levels of tracheal antimicrobial peptides (TAP), bovine tongue-derived lingual antimicrobial peptide (LAP), epithelial cell-specific beta defensin (DEFB)1, and bovine neutrophil β-defensins (BNBD-4,5,10) AMP genes were upregulated in *S. aureus*-infected MAC-T cells compared to untreated controls (Fig. 4). Taken together, these findings suggest that safer dosage of OA can induce AMP gene expression under in vivo conditions promoting the bacterial clearance.

### Membrane damage, serial passage, and survival assay

**Fig. 5:**
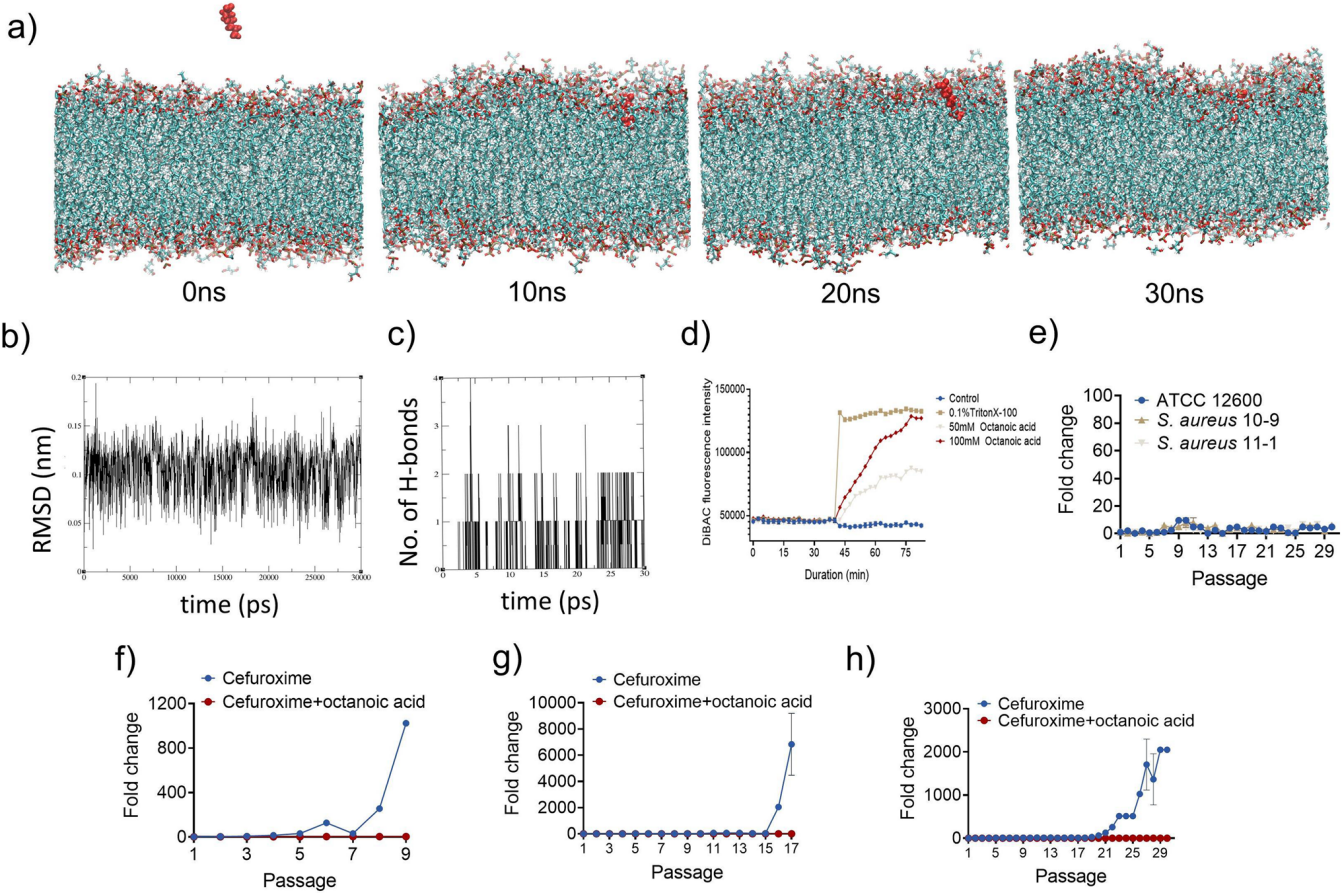
Membrane damage and developed drug resistance assay. **a** Interaction of OA with Gram-positive membrane using molecular dynamics; **b** H-bond formation; **c** root-mean squared deviation; **d** DiBAC membrane potential fluorescent assay; **e** Development of drug resistance by *S. aureus* ATCC 12600, *S. aureus* 10-9, *S. aureus* 11-1 when treated with OA. Development of drug resistance after treatment with cefuroxime or cefuroxime with OA in *S. aureus* ATCC 12600 (**f**), *S. aureus* 10-9 (**g**), *S. aureus* 11-1 (**h**)

Molecular dynamics simulations were performed to identify the membrane interaction potential of OA against Gram-positive membranes. In the simulations, snapshots were captured every 10 ns (Fig. 5a). The results showed that OA can attach to the membrane in less than 10 ns and penetrate inside the membrane. At 30 ns, a deformity in the membrane was observed, which could further lead to membrane damage. Root mean squared deviations (RMSD) were observed to be lower than 0.25 nm, and H-bond formation (Fig. 5c) indicates a stable interaction of the OA molecule with the Gram-positive membranes (Fig. 5b). To validate that OA can cause membrane damage, fluorescent spectroscopy analysis was performed using the bis-(1,3-dibutylbarbituric acid)trimethine oxonol (DiBAC) fluorophore, which can indicate membrane depolarization potential. The fluorescence was increased in a concentration-dependent manner upon treatment with OA at 50 mM and 100 mM. This result suggests that OA compromises membrane integrity and induces depolarization (Fig. 5d).

Given the ongoing concern of induced drug resistance, we assessed the ability of *S. aureus* to develop resistance to long-term exposure of OA and the antibiotic cefuroxime. Serial passaging of *S. aureus* in the presence of sub-inhibitory concentrations of OA resulted in a very low incidence of resistance development, with less than a tenfold increase in MIC even after 30 passages (Fig. 5e). In contrast, long-term exposure of *S. aureus* ATCC 12600 to cefuroxime resulted in a significant 1024-fold increase in MIC after 9 passages (Fig. 5f). After 16 passages of *S. aureus* 10-9, a 2048-fold increase in MIC was observed (Fig. 5g). After 26 passages in *S. aureus* 11-1, an increase in MIC of over 1024-fold was seen (Fig. 5h). However, continuous exposure of these *S. aureus* strains to the antibiotic cefuroxime in combination with 4 mM OA prevented the development of antibiotic resistance. Over the course of 30 passages, the MIC for cefuroxime in all *S. aureus* strains increased by no more than fourfold when OA was present. This result shows that OA can potentially be used in combination with commercial antibiotics to help prevent drug resistance development. Figure S4 shows the survival of *Galleria mellonella* upon administration of a high dosage of OA. Survival was observed to be 100% up to 1000 mM and reduced to 50% at 2000 mM.

### Transcriptome analysis

**Fig. 6:**
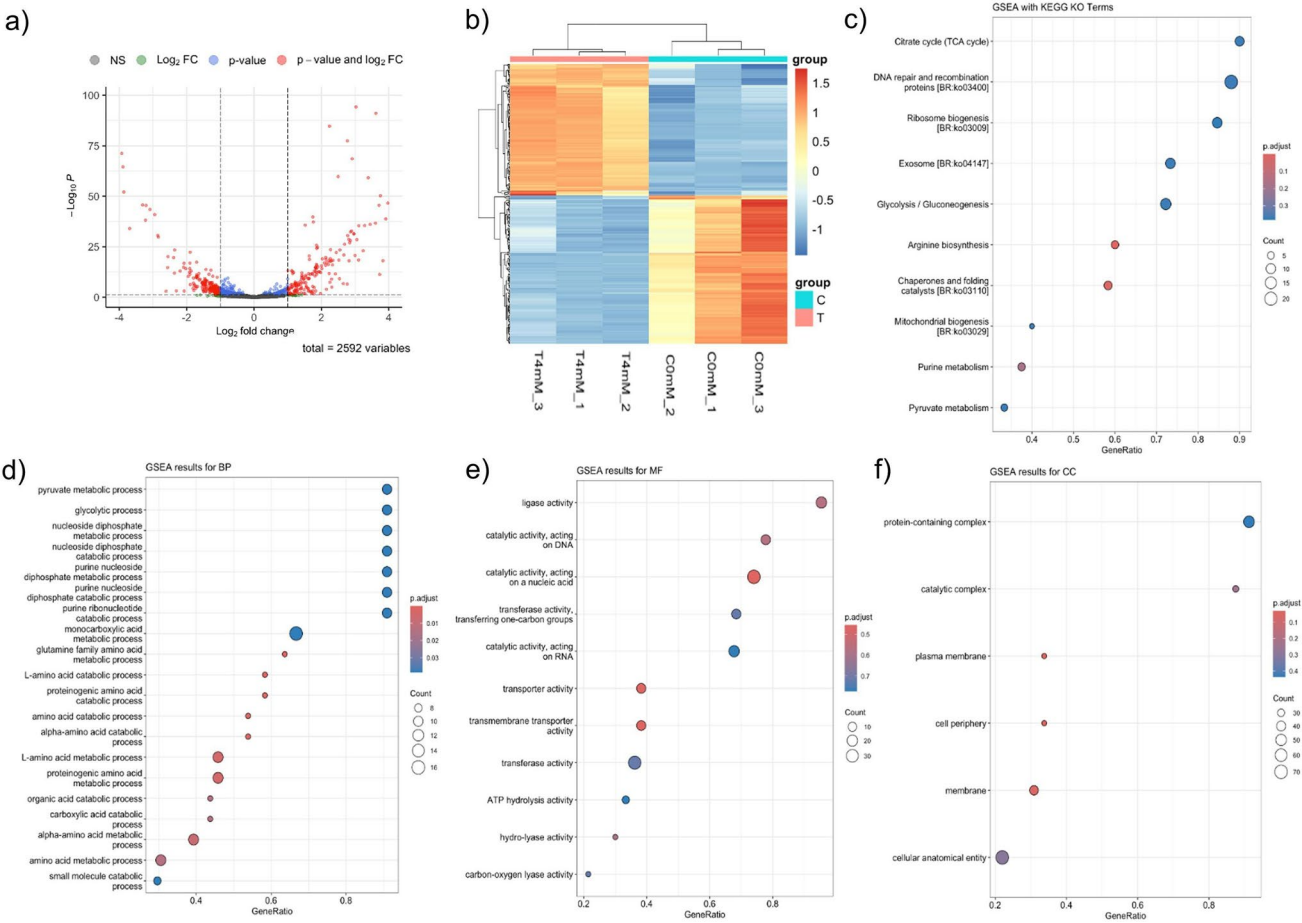
Transcriptome analysis. **a** Volcano plots for DEGs. **b** Heatmap of significant DEGs [(Padj < 0.05; |log2 fold change|≥ 1); Z-scores, TMM normalized]. **c** Gene-set enrichment analysis (GSEA) with KEGG KO terms; **d** GSEA results for biological process (BP); **e** GSEA results for molecular function (MF); **f** GSEA results for cellular components (CC)

High-throughput RNA sequencing (RNA-seq) was conducted to investigate the transcriptional mechanisms underlying the action of OA; analyses were performed in triplicate. Global transcriptomic analyses of antimicrobial responses commonly use sub-inhibitory or sub-lethal concentrations to capture stress responses and regulatory adaptations without complete bacterial eradication. Therefore, a 4-mM treatment was applied to achieve a sub-lethal or early-lethal state in *S. aureus* 13-1 (Popella et al. 2021). The overall alignment rate of the obtained reads exceeded 90%, which is significantly higher than the standard threshold of 70%. This high alignment rate confirmed the high quality of clean reads and the suitability of the reference genome for downstream analyses (Table S3). TPM density plots were made to show the distribution of normalized gene expression across samples, while box plots are used to demonstrate uniform expression levels in a quartile representation (Figure S5). Principal component analysis (PCA) showed there was 18.7% variation between the untreated and treated sample groups (Figure S6). A total of 2592 DEGs were identified as regulated by OA treatment. Among the identified candidates, 168 genes were significantly upregulated and 396 genes were significantly downregulated (Padj < 0.05; |log2 fold change|≥ 1). Volcano plots of the DEGs and a heatmap showing significant DEGs are presented in Fig. 6a and 6b.

### KEGG pathway and GO enrichment analysis

We next wanted to understand the biological pathways affected by OA. The Kyoto Encyclopedia of Genes and Genomes (KEGG) is a highly integrated database that offers insights into biological systems and their molecular, cellular, and organism-level interactions. The DEGs were analyzed for pathway enrichment, and the most significantly enriched pathways are displayed in Fig. 6c. The top five enriched pathways included the TCA cycle, DNA repair and recombination proteins, ribosome biosynthesis, exosome processes, and glycolysis/gluconeogenesis.

Furthermore, Gene Ontology (GO) analysis is an internationally standardized system for classifying gene functions. Thus, GO analysis was performed to explore the properties and roles of genes and gene products associated with DEGs between the OA-treated and control groups. The analysis revealed 20 terms associated with biological processes (BPs) (Fig. 6d), 11 terms for molecular functions (MFs) (Fig. 6e), and 6 terms related to cellular components (CCs) (Fig. 6f). The top five enriched BP categories were pyruvate metabolic process, glycolytic process, nucleotide diphosphate metabolic process, nucleotide diphosphate catabolic process, and purine nucleoside. For MF categories, the top five were ligase activity, acting on DNA (catalytic), acting on nucleic acid (catalytic), transferase activity, and acting on RNA (catalytic). For CC categories, the top five were targeting protein-containing complex, catalytic complex, plasma membrane, cell periphery, and membrane.

### Differentially expressed gene (DEG) analysis

**Fig. 7:**
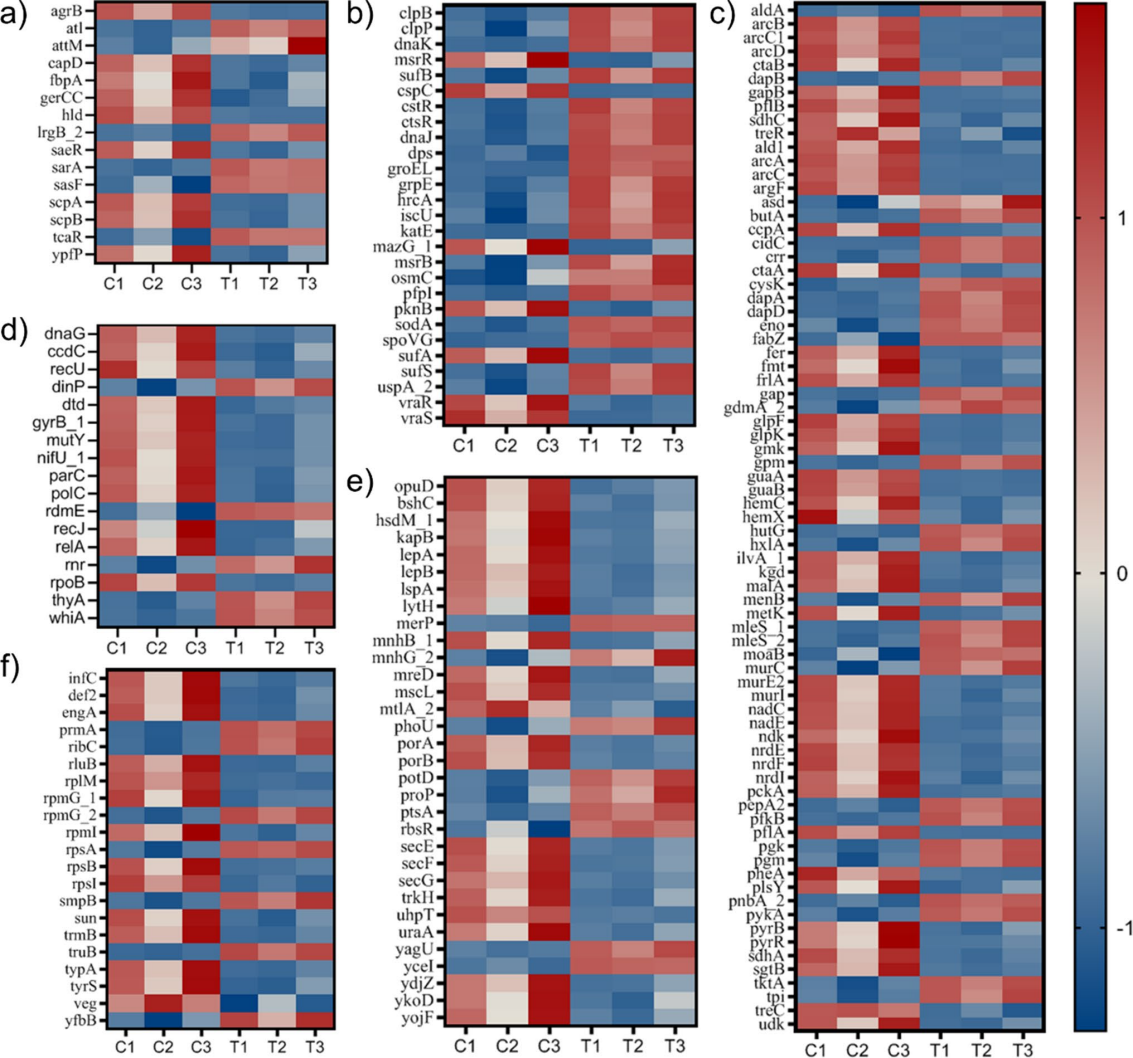
DEG analysis of significantly regulated genes (Padj < 0.05; |log2 fold change|≥ 1). **a** Virulence factors; **b** stress response and adaptation; **c** metabolic enzymes; **d** DNA replication, repair, and recombination; **e** transporters and membrane proteins; **f** ribosomal proteins and translation

Transcriptomic analysis of *S. aureus* treated with octanoic acid (OA) revealed significant alterations in gene expression across multiple functional categories, including virulence factors, metabolic enzymes, stress response pathways, DNA replication and repair, ribosomal proteins, and membrane-associated transporters. These changes indicate a broad transcriptional response of *S. aureus* to OA exposure. Among virulence-associated genes (Fig. 7a), the fbpA gene, which encodes fibronectin-binding protein A involved in bacterial adhesion to host epithelial and endothelial cells, was significantly downregulated following OA treatment. In addition, the expression of agrB, a key component of the accessory gene regulator (*agr*) quorum-sensing system, was markedly altered. Given the central role of the *agr* system in coordinating virulence gene expression, these changes suggest a potential perturbation of *agr*-mediated regulatory networks. Furthermore, genes such as atl, involved in cell wall remodeling, and lrgB_2, which modulates murein hydrolase activity and biofilm-associated processes, also exhibited significant differential expression. A comprehensive list of differentially expressed virulence-related genes and their predicted functions is provided in Table S4.

Genes involved in stress response and environmental adaptation were also significantly affected by OA treatment (Fig. 7b; Table S5). The ATP-dependent proteases clpB and clpP, which play roles in protein quality control, along with cspC, a cold-shock protein associated with stress adaptation, showed altered expression. Several heat-shock proteins, including dnaJ, dnaK, groEL, and grpE, which function as molecular chaperones to maintain protein folding under stress conditions, were differentially regulated. In addition, dps, a DNA-binding protein that protects genomic DNA during stress, and antioxidant-related genes such as katE, sodA, msrB, and msrR, which are involved in detoxifying reactive oxygen species and repairing oxidative damage, were affected. The two-component regulatory system vraR/vraS, known to mediate cell wall stress responses, also exhibited altered expression. Collectively, these transcriptional changes are consistent with the induction of a stress-response program in *S. aureus* upon OA exposure.

OA treatment also extensively impacted genes involved in metabolic pathways, particularly those associated with carbohydrate metabolism and amino acid biosynthesis (Fig. 7c; Table S6). Differential expression of ctaA and ctaB, which are involved in heme A biosynthesis for the electron transport chain, suggests potential modulation of respiratory processes. The altered expression of cidC, encoding pyruvate oxidase, and arcA, arcB, and arcC, components of the arginine deiminase pathway, indicates a possible metabolic shift under OA-induced stress conditions. Additionally, genes such as fabZ, involved in fatty acid biosynthesis, and gpm, a key enzyme in glycolysis, were dysregulated, suggesting interference with central metabolic pathways. Concurrently, the upregulation of stress-related chaperone genes (dnaJ, dnaK, and groEL) further supports the notion that OA induces metabolic and proteotoxic stress. The downregulation of sufA and sufB, which are required for iron–sulfur cluster assembly, suggests that OA treatment may promote oxidative stress conditions that affect essential cofactor biosynthesis.

Genes associated with DNA replication, repair, and recombination were also significantly affected by OA treatment (Fig. 7d; Table S7). Core replication genes such as dnaG (DNA primase) and polC (DNA polymerase III subunit) displayed altered expression, indicating potential perturbations in DNA replication processes. Changes in the expression of parC (topoisomerase IV subunit) and gyrB_1 (DNA gyrase subunit) suggest possible interference with DNA supercoiling and chromosome segregation. In addition, genes involved in DNA repair pathways were impacted; for example, mutY, a DNA glycosylase involved in base excision repair, was downregulated, which may reduce the efficiency of oxidative DNA damage repair. Similarly, reduced expression of recombination-associated genes recJ and recU suggests potential impairment of homologous recombination-mediated repair. The downregulation of relA, a regulator of the stringent response, further indicates that OA may affect global stress adaptation mechanisms. Together, these transcriptional changes suggest that OA exposure may compromise pathways involved in genomic maintenance and stress resilience.

The expression of genes associated with cell membrane integrity and transport functions was also altered following OA treatment (Fig. 7e; Table S8). The gene lytH, which regulates autolysin activity and contributes to cell wall turnover, exhibited differential expression, suggesting possible effects on cell envelope remodeling. The mechanosensitive channel gene mscL, which enables bacterial cells to respond to membrane tension, was also affected. In addition, porA and porB, encoding porins involved in nutrient and ion transport, showed altered expression, indicating potential disruption of membrane transport processes. Transporters such as potD and proP, which contribute to osmotic stress adaptation, were similarly dysregulated. These findings collectively suggest that OA treatment may impose stress on membrane homeostasis and transport systems in *S. aureus*.

OA treatment further influenced the expression of genes encoding ribosomal proteins and components of the translational machinery (Fig. 7f; Table S9). Differential expression of small ribosomal subunit proteins (rpsA, rpsB, and rpsI) and the large subunit protein rplM suggests potential perturbations in ribosome assembly and protein synthesis. The translation-associated GTPase typA, which modulates translational efficiency under stress conditions, was upregulated, possibly reflecting an adaptive response to translational stress. In addition, altered expression of truB and rluB, which are involved in pseudouridine modification of tRNA and rRNA, indicates potential effects on translation fidelity and ribosome stability. The downregulation of veg, a regulator of translation, further suggests a global adjustment of protein synthesis in response to OA exposure. Overall, these transcriptional changes indicate that OA treatment elicits a multifaceted stress response that affects multiple essential physiological processes in *S. aureus*.

### Density function theory and physicochemical properties

**Fig. 8:**
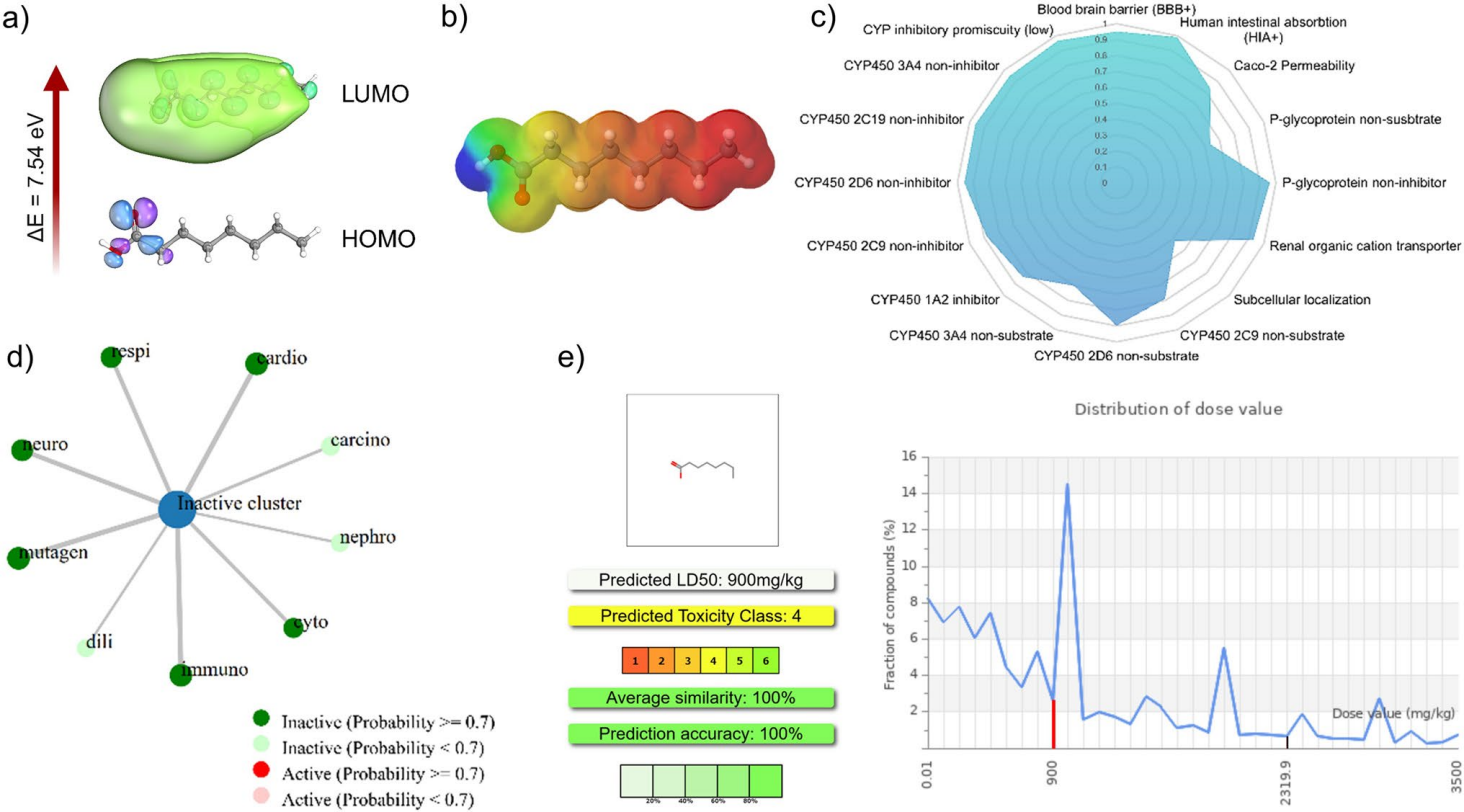
Quantum chemical and physicochemical properties of OA. **a** Optimized structure of OA; **b** DFT calculation analysis; **c** ADME predictions; **d** toxicity predictions; **e** toxicity profile predictions

The quantum chemical properties of octanoic acid (OA) were evaluated using density functional theory (DFT) with the B3LYP/6-311G + + (d,p) functional following energy minimization (Figure S7; Table S10). The calculated highest occupied molecular orbital (HOMO, − 7.82 eV) and lowest unoccupied molecular orbital (LUMO, − 0.27 eV) energies provide insight into the electronic characteristics of OA (Fig. 8a). The relatively large HOMO–LUMO energy gap (7.54 eV) suggests high electronic stability and low intrinsic chemical reactivity, indicating a reduced tendency for spontaneous electron transfer reactions. Such electronic stability is generally considered a favorable property in drug-like molecules, as it may lower the likelihood of nonspecific chemical interactions.

Based on hard–soft acid–base (HSAB) theory (Pearson 2005; Eunice et al. 2025), additional global reactivity descriptors were derived to further assess the predicted chemical behavior of OA. The calculated chemical hardness (*η* = 3.77 eV) and softness (*S* = 0.26 eV) indicate resistance to electronic deformation and limited polarizability during molecular interactions. These features suggest a relatively rigid molecular framework with constrained reactivity. The predicted ionization energy (7.82 eV) reflects a strong tendency to retain electrons, while the electron affinity (0.27 eV) indicates a limited propensity to accept additional electrons. Together, these parameters are consistent with a chemically stable and moderately reactive molecule.

The chemical potential (*µ* = 4.04 eV) and electronegativity (*χ* = − 4.04 eV) further describe the predicted electron-attracting ability of OA, suggesting a balanced electronic character that may support selective molecular interactions. The electrophilicity index (*ω* = 2.17 eV) indicates a moderate tendency to participate in electrophilic interactions, which may be advantageous for controlled interactions with biological targets. The electrostatic potential (ESP) map highlights the electron-rich and electron-deficient regions of OA, providing a visual representation of potential interaction sites (Fig. 8b).

The physicochemical properties of OA were further assessed using in silico prediction tools (Table S11). OA exhibited a molecular weight of 144.21 g/mol, a topological polar surface area (TPSA) of 37.30 Å^2^, six rotatable bonds, one hydrogen bond donor, and two hydrogen bond acceptors. The predicted lipophilicity values (iLOGP = 1.95 and MLOGP = 1.96) indicate moderate lipophilicity. OA complies with Lipinski’s Rule of Five, suggesting favorable drug-likeness properties. However, the predicted low aqueous solubility (Log S = − 2.26) indicates that formulation strategies may be required to enhance bioavailability.

In silico ADME/Tox analysis was performed to evaluate the predicted absorption, distribution, metabolism, excretion, and toxicity profile of OA (Fig. 8c–e). The analysis indicated favorable ADME characteristics and did not predict major toxicity alerts under the applied models. The predicted median lethal dose (LD₅₀) was approximately 900 mg/kg, classifying OA within toxicity class 4 according to the prediction tool used. These computational results provide supportive insights into the chemical and pharmacokinetic properties of OA; however, experimental validation will be required to confirm its biological behavior and safety profile.

## Discussion

The impact of agricultural antimicrobial use on human health remains insufficiently understood. Nevertheless, agricultural applications account for approximately 44% of antibiotic usage by weight in the UK and an estimated 70% in the USA. Research has demonstrated that agricultural antimicrobial usage can greatly influence the human resistome (Lanyon et al. 2021). Notably, the use of colistin, a critical last-resort antibiotic, has been linked to the transfer of the resistance gene mcr-1 from agricultural settings to human populations (Liu et al. 2016). Thus, there is an urgent need for comprehensive studies to investigate how farming practices and antibiotic use contribute to the development of antimicrobial resistance (AMR) in both agricultural and surrounding environments.

Regarding bovine mastitis, chronic and recurrent infections may frequently be attributed to intracellular drug-resistant bacteria. Once inside the host cells, the bacteria are largely sheltered from the effects of drugs and the immune system. This process of intracellular concealment is achieved through bacterial adherence and internalization into the host cells (Yang and Ji 2014). Our study clearly shows that OA treatment can prevent adhesion and internalization of *S. aureus.* Table S12 shows the features and findings of our study compared to previous studies, highlighting the novelty and significance (Nair et al. 2005; Lin et al. 2023; Balta et al. 2024). Molecular docking studies further show that OA has the potential to inhibit adhesion proteins through direct binding. Docking analysis of OA revealed the formation of two hydrogen bonds with Clumping factor B, a protein involved in bacterial adherence, at THR266 and GLY269 in the binding site domain (Talukdar et al. 2016). Similarly, OA formed two hydrogen bonds at THR229 and LEU328 residues in Fibronectin-binding protein (FnBP), which facilitates host cell attachment. Sortase, a protein crucial for anchoring surface proteins, exhibited two hydrogen bonds at ARG197 (vital for substrate binding) and one at CYS184 (enzyme catalytic site) (Tian and Eriksson 2011). Adhesion and internalization mechanisms differ across bacterial species because each pathogen expresses distinct sets and levels of surface adhesion proteins, and MAC-T cells present varying host receptors. Therefore, OA is unlikely to act through a single conserved pathway, and its inhibitory magnitude naturally varies among *S. aureus*,* E. coli*, and *Streptococcus* spp. The inflammatory conditions in bovine mastitis are largely caused by the release of toxins by the pathogens. These toxins can destroy the mammary gland tissue and reduce milk quality. It is therefore essential for a therapy to reduce inflammation while also combating the infection. Neutralizing bacterial toxins via effects on regulatory genes or direct inhibition could be possible mechanisms of OA to reduce virulence. OA displayed stable hydrogen bond formation at SER72 and TYR80 residues on the α-hemolysin monomer and four hydrogen bonds at LYS58, TYR102, THR145, and ASP227 on γ-hemolysin monomers, demonstrating its inhibitory potential to prevent heptameric pore assembly. Additionally, three hydrogen bonds were observed at SER203**,** TYR159, and GLY204 against the superantigen Enterotoxin B. Similarly, two hydrogen bonds were identified against the Toxic Shock Syndrome Toxin and Exfoliative Toxin B, indicating the toxin-neutralizing potential of OA. Along these lines, OA has been shown to mitigate toxicity in vitro and bind to toxins and monomers that could possibly affect the assembly of pores.

In response to pathogen exposure, mammary cells trigger host defense mechanisms that upregulate the expression of AMPs to fight against invading pathogens. Host AMPs, such as tracheal antimicrobial peptides (TAP), bovine tongue-derived Lingual antimicrobial peptide (LAP), epithelial cell-specific beta defensin (DEFB)1, and bovine neutrophil β-defensin (BNBD), are known to directly kill pathogens or neutralize exogenous toxic substances (Kharayat et al. 2019; Z. Zhang et al. 2021; Shinozuka et al. 2022; Neumann et al. 2023). OA activates host defense mechanisms by upregulating AMP genes that could participate in multifaceted targeting of the pathogens.

The OA concentration used in this study was chosen based on specific biological objectives and constraints. For bactericidal activity in milk, OA was applied at 50 mM, which corresponds to the effective concentration required for medium-chain fatty acids to exert rapid antimicrobial effects under a protein-rich complex matrix like milk and a higher bacterial load of 10⁷ CFU/mL. Global transcriptome studies of antimicrobial responses generally use either sub-inhibitory/sub-lethal concentrations of the antibiotic of interest to capture stress responses and regulatory adaptations without complete bacterial eradication (Popella et al. 2021). Therefore, a 4-mM treatment was applied to induce a sub-lethal or early-lethal state in *S. aureus* 13-1. For host-cell–based assays, such as adhesion, internalization, and gene expression analysis on MAC-T cells, OA concentrations ranging from 0.5 to 4 mM were used. Importantly, this range showed no significant cytotoxicity to MAC-T cells compared with untreated controls (Fig. S3).

We found that OA was likely to damage the integrity of the membranes, according to molecular dynamics simulation and fluorescence assays. Recent studies have indicated a connection between the recurrent mastitis in dairy cows and chronic infections. Therefore, the complete eradication of invading pathogens during early stages of infection is highly beneficial, as it can prevent the development of chronic infections. Bovine mastitis is typically not caused by a single pathogenic microorganism, and some pathogens may respond poorly to current treatments. Single or combined antibiotic therapies are widely applied to treat bovine mastitis in commercial farms (Morales-Ubaldo et al. 2023). However, the pathogenic bacteria often exhibit evasion mechanisms that make antibiotic therapy inefficient (Fisher et al. 2017). Nevertheless, OA could clear pathogens more quickly than antibiotics, and OA did not induce resistance in serial passage assays. Impressively, *S. aureus* did not develop resistance against OA, even after 30 passages, and the combination of OA with antibiotics helped to prevent the development of resistance. These findings suggest that OA may be utilized as a single treatment or in combination with commercial antibiotics.

Transcriptomic analysis of *S. aureus* treated with OA revealed extensive alterations in multiple functional categories. A wide range of virulence genes, stress response genes, metabolic genes, DNA replication and repair genes, membrane integrity, and nutrient transport genes were significantly altered.

These findings suggest that OA induces a broad, multi-level disruption of *S. aureus* physiology. Furthermore, OA exhibits favorable quantum chemical properties, including a large HOMO–LUMO gap (10.96 eV), which indicates high chemical stability and low reactivity. Such properties are typically beneficial for drug safety. The moderate electronegativity and electrophilic index for OA suggest a potential for selective interactions with biological targets. Physiochemically, OA complies with Lipinski’s Rule of Five, indicating drug-likeness, though its low water solubility may require formulation adjustments to improve bioavailability. The ADME/Tox analysis revealed no significant toxicity, with an LD50 of 900 mg/kg. Thus, OA can be classified as a relatively safe compound for therapeutic use.

## Conclusion

This study integrates microbiological assays, transcriptomic analysis, molecular modeling, and in silico chemical evaluations to investigate the antimicrobial potential and mode of action of octanoic acid. OA effectively reduced *S. aureus* adhesion and internalization into epithelial cells, neutralized bacterial toxicity, activated host defense responses through upregulation of AMP genes, disrupted bacterial membrane integrity, and exhibited a low propensity for resistance development even after prolonged exposure. Transcriptomic profiling revealed widespread alterations in gene expression associated with virulence regulation, stress adaptation, metabolism, DNA maintenance, and membrane-associated processes, suggesting that OA elicits a broad and coordinated bacterial stress response. Computational analyses further predicted favorable chemical stability, physicochemical properties, and ADME/Tox characteristics, supporting the drug-likeness of OA, although these findings remain predictive in nature.

While OA demonstrates strong antimicrobial efficacy and multimodal activity in vitro, supported by transcriptomic and in silico analyses, further studies are required to validate these mechanisms at the protein and functional levels. In particular, comprehensive in vivo investigations are necessary to determine optimal dosing, long-term safety, pharmacokinetics, pharmacodynamics, and host–pathogen interactions. Overall, this work highlights the potential of screening naturally occurring multimodal molecules as a rational strategy for developing smarter antimicrobial agents to combat persistent infections and antimicrobial resistance.

## Supplementary Information

Below is the link to the electronic supplementary material.

ESM 1 (DOCX 3.26 MB)

## Data Availability

The triplicates of the RNA-Seq raw data were deposited as BioProject into the National Centre for Biotechnology Information database under accession number [SRR34065388](https://dataview.ncbi.nlm.nih.gov/object/SRR34065388?reviewer=n1moj530ch5b4uqriqiorf7dlt) (C0mM_1), [SRR34065387] (https://dataview.ncbi.nlm.nih.gov/object/SRR34065387?reviewer=n1moj530ch5b4uqriqiorf7dlt) (C0mM_2), [SRR34065386] (https://dataview.ncbi.nlm.nih.gov/object/SRR34065386?reviewer=n1moj530ch5b4uqriqiorf7dlt) (C0mM_3), [SRR34065385](https://dataview.ncbi.nlm.nih.gov/object/SRR34065385?reviewer=n1moj530ch5b4uqriqiorf7dlt) (T4mM_1), [SRR34065384] (https://dataview.ncbi.nlm.nih.gov/object/SRR34065384?reviewer=n1moj530ch5b4uqriqiorf7dlt) (T4mM_2), [SRR34065383] (https://dataview.ncbi.nlm.nih.gov/object/SRR34065383?reviewer=n1moj530ch5b4uqriqiorf7dlt) (T4mM_3); and are available at the following URL: [https://dataview.ncbi.nlm.nih.gov/object/PRJNA1279634?reviewer=n1moj530ch5b4uqriqiorf7dlt] (https://dataview.ncbi.nlm.nih.gov/object/PRJNA1279634?reviewer=n1moj530ch5b4uqriqiorf7dlt).
