# Supplementary material for: Multimodal molecular mechanisms of octanoic acid (OA) against recurrent mastitis causing pathogens
Source: Appl Microbiol Biotechnol. 2026 Feb 5;110(1):58. doi: 10.1007/s00253-026-13727-y (PMC12881107; doi:10.1007/s00253-026-13727-y)
Supplement: Supplementary file 1 — (DOCX 3.26 MB) [file 253_2026_13727_MOESM1_ESM.docx]

**Multimodal molecular mechanisms of octanoic acid (OA) against recurrent mastitis causing pathogens**

Kai-Chen Hsu^a‡^, Sanjay Prasad Selvaraj^b,c‡^, Ming-Feng You^d^, Wen-Chun Lin^d^, Tsai-Ming Lu^g^, Kuo-Hua Lee^e*^, Chau-Hwa Chi^a*^, Jyh-Yih Chen^d,f*^

*^a^ Department of Veterinary Medicine, National Taiwan University, Taipei, Taiwan*

*^b^ Molecular and Biological Agricultural Science Program, Taiwan International Graduate Program, Academia Sinica, Taipei, 11529, Taiwan;*

*^c^ Graduate Institute of Biotechnology, National Chung Hsing University, Taichung, 402, Taiwan.*

*^d^ Marine Research Station,* *Institute of Cellular and Organismic Biology, Academia Sinica, 23-10 Dahuen Road, Jiaushi, Ilan, 262, Taiwan*

*^e^ Northern Region Branch, MOA-TRI, Miaoli, Taiwan*

*^f^ iEGG and Animal Biotechnology Center and the Rong Hsing Research Center for Translational Medicine, National Chung Hsing University, Taichung 402, Taiwan*

*^g^ Institute of Cellular and Organismic Biology, Academia Sinica, Nankang 115201, Taiwan*

*Corresponding author at: Marine Research Station, Institute of Cellular and Organismic Biology, Academia Sinica, 23-10 Dahuen Rd. Jiaushi, Ilan 262, Taiwan, Tel.: 886-920802111, Fax: 886-39871035; email: zoocjy@gate.sinica.edu.tw (Jyh-Yih Chen)

*Corresponding author at: Department of Veterinary Medicine, National Taiwan University, Taipei, Taiwan (No. 1, Sec. 4, Roosevelt Road, Taipei City, Taiwan); e-mail: [chie@ntu.edu.tw](mailto:chie@ntu.edu.tw) (Chau-Hwa Chi)

*Corresponding author at: Northern Region Branch, MOA-TRI, Miaoli, Taiwan (No. 207-5, Bi-tou-mian, Wu-hoo village, Si-hoo Township, Miaoli County, Taiwan); e-mail: [khlee@mail.tlri.gov.tw](mailto:khlee@mail.tlri.gov.tw) (Kuo-Hua Lee)

‡These authors contributed equally.

**Table S1: Bacterial isolates used in this study**

| **Reference bacterial strain** | | | |
| --- | --- | --- | --- |
| ATCC 12600 *Staphylococcus aureus* | | |  |
| **Clinical isolates from raw milk derived from dairy cows with mastitis** | | | |
| **Isolate** | **Recurrent mastitis** | **Species** |  |
| ***Streptococcus* sp.** | | |  |
| 1-1 |  | *Streptococcus* sp. |  |
| 2-1 |  | *Streptococcus equinus* |  |
| 2-6 |  | *Streptococcus lutetiensis* |  |
| 3-2 |  | *Streptococcus equinus* |  |
| 6-2 |  | *Streptococcus uberis* |  |
| 9-6 | R | *Streptococcus equinus* |  |
| ***Staphylococcus* sp**. | | |  |
| 7-1 |  | *Staphylococcus haemolyticus* |  |
| 7-2 |  | *Staphylococcus aureus* |  |
| 7-3 |  | *Staphylococcus aureus* |  |
| 10-9 | R | *Staphylococcus aureus* |  |
| 11-1 | R | *Staphylococcus aureus* |  |
| 12-12 | R | *Staphylococcus aureus* |  |
| 13-1 | R | *Staphylococcus aureus* |  |
| ***Escherichia coli*** | | |  |
| 7-13 |  | *Escherichia coli* |  |
| 8-8 | R | *Escherichia coli* |  |
| 9-8 | R | *Escherichia coli* |  |
| ***Enterococcus faecium*** | | |  |
| 5-1 |  | *Enterococcus faecium* |  |
| 8-3 | R | *Enterococcus faecium* |  |

The clinical isolate number comprises the udder identifier followed by the pathogen strain number. Bovine udders numbered 8 to 13 showed recurrent clinical mastitis. R denotes drug-resistant strains

**Table S2. List of primers used in this study.**

| **Gene** | **Oligonucleotide (5′-3′)** | **Amplicon size (bp)** | **GenBank** | | |
| --- | --- | --- | --- | --- | --- |
| **Bovine mammary epithelial cell** | | | | | |
| Bovine neutrophil β-defensin 5 (BNBD5) | F: GCCAGCATGAGGCTCCATC | 143 | AF014108.1 | | |
|  | R: TTGCCAGGGCACGAGATCG |  |  |  |  |
| Bovine lingual antimicrobial peptide (LAP) | F: GCCAGCATGAGGCTCCATC | 194 | NM_203435.4 | | |
|  | R: CTCCTGCAGCATTTTACTTGGG |  |  |  |  |
| Bovine neutrophil β-defensin 10 (BNBD10) | F: GCTCCATCACCTGCTCCTC | 152 | NM_001115084.1 | | |
|  | R: AGGTGCCAATCTGTCTCATGC |  |  |  |  |
| Bovine neutrophil β-defensin 4 (BNBD4) | F: GCCAGCATGAGGCTCCATC | 278 | NM_174775.1 | | |
|  | R: CGTTTAAATTTTAGACGGTGT |  |  |  |  |
| Bovine β-defensin 1 (DEFB1) | F: CCATCACCTGCTCCTCACA | 185 | BC114788.1 | | |
|  | R: ACCTCCACCTGCAGCATT |  |  |  |  |
| Bovine tracheal antimicrobial peptide (TAP) | F: GCGCTCCTCTTCCTGGTCCTG | 217 | NM_174776.1 | | |
|  | R: GCACGTTCTGACTGGGCATTGA |  |  |  |  |
| β-actin | F: CCTTTTACAACGAGCTGCGTGTG | 391 | AH001130.2 | | |
|  | R: ACGTAGCAGAGCTTCTCCTTGATG |  |  |  |  |
|  | R: ATTACCGCGGCTGCTGG |  |  |  |  |
| F: forward, R: reverse |  |  | |  |  |

**Table S3:** **Total mapping rate of the samples**

| **Sample** | **Mapping Rate (%)** |
| --- | --- |
| Control 1 | 97.48 |
| Control 2 | 97.77 |
| Control 3 | 97.51 |
| Treated 1 | 98.11 |
| Treated 2 | 98.20 |
| Treated 3 | 98.15 |

**Table S4: Differential genes regulating virulence factors**

| **Gene name** | **Functions** |
| --- | --- |
| agrB | Accessory gene regulator system component involved in quorum sensing and virulence regulation. |
| atl | Autolysin, involved in cell wall remodeling |
| attM | Involved in quorum sensing signal degradation |
| capD | Capsule biosynthesis protein |
| fbpA | Potential virulence factor as it may play a role in adherence |
| gerCC | Germination protein, linked to spore germination (may indirectly affect virulence) |
| hld | Encodes δ-hemolysin, a toxin contributing to virulence. |
| lrgB_2 | Regulates murein hydrolase activity and affects biofilm formation |
| saeR | Part of the SaeRS two-component system regulating virulence genes |
| sarA | Global regulator of virulence factor expression. |
| sasF | Surface-associated virulence factor |
| scpA, scpB | Segregation proteins, part of the virulence regulation system |
| tcaR | Transcriptional regulator of antibiotic resistance |
| ypfP | Synthesizes glycolipids, which are often involved in virulence |

**Table S5: Differential genes regulating stress response and adaptation**

| **Gene name** | **Functions** |
| --- | --- |
| clpB, clpP | ATP-dependent proteases involved in protein quality control under stress |
| cspC | Cold-shock protein |
| cstR | Regulator of stress response. |
| ctsR | Controls heat shock response by regulating molecular chaperones |
| dnaJ, dnaK | Molecular chaperones involved in protein folding and stress response |
| dps | DNA-binding protein protecting DNA under stress conditions |
| groEL | Heat shock protein chaperone |
| grpE | Co-chaperone involved in protein folding |
| hrcA | Regulator of heat shock proteins |
| iscU | Iron-sulfur cluster scaffold protein |
| katE | Catalase involved in hydrogen peroxide detoxification |
| mazG_1 | Nucleotide metabolism regulator linked to stress responses |
| msrB, msrR | Methionine sulfoxide reductases for oxidative stress repair |
| osmC | Osmotically inducible protein involved in stress response |
| pfpI | Involved in stress responses, protease activity |
| pknB | Serine/threonine-protein kinase, involved in signaling under stress conditions |
| sodA | Superoxide dismutase involved in reactive oxygen species detoxification |
| spoVG | Regulator of sporulation |
| sufA, sufB, sufS | Iron-sulfur cluster assembly proteins, crucial under oxidative stress conditions |
| uspA_2 | Universal stress protein |
| vraR, vraS | Two-component system for cell wall stress response |

**Table S6: Differential genes regulating metabolic enzymes**

| **Gene name** | **functions** |
| --- | --- |
| ald1 | Involved in aldehyde metabolism |
| aldA | Aldehyde dehydrogenase involved in aldehyde metabolism |
| arcA, arcB, arcC1, arcD | Arginine deiminase pathway enzymes involved in arginine catabolism |
| arcC | Arginine catabolism enzyme (arc operon component) |
| argF | Encodes ornithine carbamoyltransferase, involved in the urea cycle |
| asd | Aspartate-semialdehyde dehydrogenase, involved in amino acid biosynthesis |
| butA | Butyrate kinase involved in butyrate metabolism |
| ccpA | Catabolite control protein, regulates carbon metabolism |
| cidC | Pyruvate oxidase involved in cell death and biofilm development |
| crr | Part of the phosphotransferase system, carbohydrate metabolism |
| ctaA, ctaB | Cytochrome c oxidase assembly proteins involved in heme A biosynthesis |
| cysK | Cysteine synthase involved in cysteine biosynthesis |
| dapA, dapB, dapD | Enzymes involved in diaminopimelate and lysine biosynthesis |
| eno | Enolase, a glycolytic enzyme converting 2-phosphoglycerate to phosphoenolpyruvate |
| fabZ | Enoyl-[acyl-carrier-protein] hydratase involved in fatty acid biosynthesis |
| fer | Ferredoxin, involved in electron transfer |
| fmt | Methionyl-tRNA formyltransferase, essential for initiating protein synthesis |
| frlA | Fructoselysine metabolism enzyme |
| gap, gapB | Glyceraldehyde-3-phosphate dehydrogenases involved in glycolysis and gluconeogenesis |
| gdmA_2 | Involved in guanine metabolism |
| glpF, glpK | Glycerol uptake facilitator and glycerol kinase involved in glycerol metabolism |
| gmk | Guanylate kinase, nucleotide metabolism |
| gpm | Phosphoglycerate mutase, a glycolytic enzyme |
| guaA, guaB | Enzymes involved in guanine nucleotide biosynthesis |
| hemC | Porphobilinogen deaminase involved in heme biosynthesis |
| hemX | Heme biosynthesis regulation |
| hutG | Involved in histidine utilization |
| hxlA | Formaldehyde assimilation (ribulose monophosphate pathway) |
| ilvA_1 | Threonine deaminase involved in isoleucine biosynthesis |
| kgd | α-Ketoglutarate decarboxylase in TCA cycle |
| malA | Enzyme in maltose metabolism |
| menB | Menaquinone biosynthesis enzyme |
| metK | S-adenosylmethionine synthetase involved in methionine metabolism |
| mleS_1, mleS_2 | Malate/lactate dehydrogenases |
| moaB | Molybdenum cofactor biosynthesis enzyme |
| murC, murE2, murI | Enzymes involved in peptidoglycan biosynthesis |
| nadC, nadE | Enzymes involved in NAD biosynthesis |
| ndk | Nucleoside diphosphate kinase, nucleotide metabolism |
| nrdE, nrdF, nrdI | Enzymes involved in ribonucleotide reduction for DNA synthesis |
| pckA | Phosphoenolpyruvate carboxykinase, gluconeogenesis |
| pepA2 | Peptidase activity for protein degradation |
| pfkB | Phosphofructokinase, a key glycolytic enzyme |
| pflA, pflB | Pyruvate formate-lyase and activating enzyme involved in anaerobic metabolism |
| pgk | Phosphoglycerate kinase involved in glycolysis |
| pgm | Phosphoglucomutase involved in carbohydrate metabolism |
| pheA | Prephenate dehydratase involved in phenylalanine biosynthesis |
| plsY | Glycerol-3-phosphate acyltransferase involved in phospholipid biosynthesis |
| pnbA_2 | P-nitrobenzyl esterase, involved in enzymatic reactions possibly linked to metabolism |
| pykA | Pyruvate kinase, catalyzes the final step in glycolysis |
| pyrB, pyrR | Enzymes involved in pyrimidine biosynthesis |
| sdhA, sdhC | Components of succinate dehydrogenase, part of the TCA cycle |
| sgtB | Peptidoglycan synthesis regulator |
| tktA | Transketolase involved in the pentose phosphate pathway |
| tpi | Triosephosphate isomerase involved in glycolysis |
| treC, treR | Involved in trehalose metabolism |
| udk | Uridine kinase, important for nucleotide metabolism |

**Table S7: Differential genes regulating DNA replication, repair, and recombination**

| **Gene name** | **functions** |
| --- | --- |
| ccdC | Cell division regulator in plasmid stabilization |
| dinP | DNA polymerase IV for damage tolerance |
| dnaG | DNA primase involved in DNA replication |
| dtd | D-Tyr-tRNA deacylase for quality control in translation |
| gyrB_1 | DNA gyrase subunit involved in DNA supercoiling |
| mutY | DNA glycosylase involved in DNA repair |
| nifU_1 | Iron-sulfur cluster scaffold protein, supports DNA repair enzymes |
| parC | Topoisomerase IV subunit involved in DNA replication |
| polC | DNA polymerase III subunit involved in DNA replication |
| rdmE | Role in recombination and repair processes |
| recJ, recU | Recombinational repair proteins |
| relA | Regulates (p)ppGpp synthesis during stringent response |
| rnr | Ribonucleotide reductase, critical for DNA repair and synthesis |
| rpoB | RNA polymerase subunit involved in transcription |
| thyA | Thymidylate synthase for DNA synthesis |
| whiA | Involved in DNA replication and cell division |

| **Table S8: Differential genes regulating transporters and membrane Proteins**   \| **Gene name** \| **functions** \| \| --- \| --- \| \| bshC \| Enzyme involved in bacillithiol biosynthesis \| \| glpF \| Glycerol transport protein \| \| hsdM_1 \| Part of a restriction-modification system, membrane-associated \| \| kapB \| Encodes potassium channel protein \| \| lepA \| Lipoprotein signal peptidase \| \| lspA, lepB \| Membrane proteins involved in protein transport \| \| lytH \| Autolysin regulator, contributes to cell wall turnover \| \| merP \| Mercuric ion transporter protein \| \| mnhB_1, mnhG_2 \| Membrane proteins in sodium/proton antiport systems \| \| mreD \| Cell shape-determining protein \| \| mscL \| Mechanosensitive channel protein \| \| mtlA_2 \| Mannitol-specific PTS system transporter \| \| opuD, potD \| Transporters for osmoprotectants and polyamines \| \| phoU \| Phosphate uptake regulation \| \| porA, porB \| Porins, involved in nutrient and ion transport \| \| proP \| Proline transport, related to osmotic adaptation \| \| ptsA \| Phosphotransferase system enzyme I \| \| rbsR \| Ribose transport regulatory protein \| \| secE, secF, secG \| Sec proteins involved in protein translocation \| \| trkH \| Potassium uptake transporter \| \| uhpT \| Phosphate transport system permease \| \| uraA \| Uracil permease for nucleobase uptake \| \| yagU, yceI, ydjZ, ykoD, yojF \| Likely transport-associated proteins or membrane proteins. \| |
| --- | --- | --- | --- | --- | --- | --- | --- | --- | --- | --- | --- | --- | --- | --- | --- | --- | --- | --- | --- | --- | --- | --- | --- | --- | --- | --- | --- | --- | --- | --- | --- | --- | --- | --- | --- | --- | --- | --- | --- | --- | --- | --- | --- | --- | --- | --- | --- | --- |

**Table S9: Differential genes regulating ribosomal proteins and translation**

| \| **Gene name** \| **functions** \| \| --- \| --- \| \| def2 \| Peptide deformylase primarily assists in protein synthesis \| \| engA \| GTPase involved in ribosome assembly \| \| infC \| Translation initiation factor IF-3 \| \| prmA \| Ribosomal protein methyltransferase \| \| ribC \| Translation initiation factor IF-3 \| \| rluB \| Pseudouridine synthase for tRNA and rRNA modification \| \| rplM, rpmG_1, rpmG_2, rpmI \| Ribosomal proteins involved in protein synthesis \| \| rpsA, rpsB, rpsI \| Ribosomal proteins involved in protein synthesis \| \| smpB \| Small protein binding to tmRNA, aiding stalled ribosome rescue \| \| sun \| Post-translational modification of ribosomal proteins \| \| trmB \| tRNA (guanine-N7-)-methyltransferase involved in tRNA modification \| \| truB \| tRNA pseudouridine synthase involved in tRNA modification \| \| typA \| GTPase involved in translation regulation \| \| tyrS \| Tyrosyl-tRNA synthetase involved in tRNA charging \| \| veg \| Encodes a regulatory protein for translation \| \| yfbB \| Involved in tRNA modification, which can influence translation fidelity \| |
| --- | --- | --- | --- | --- | --- | --- | --- | --- | --- | --- | --- | --- | --- | --- | --- | --- | --- | --- | --- | --- | --- | --- | --- | --- | --- | --- | --- | --- | --- | --- | --- | --- | --- | --- |

**Table S10: Density function theory (DFT) calculations**

| **Parameter** | **energy (eV)** |
| --- | --- |
| E_HOMO_ | −7.8219 |
| E_LUMO_ | −0.2756 |
| Energy gap (ΔE) | 7.5463 |
| Ionization Potential (I) | 7.8219 |
| Electron Affinity (*A*) | 0.2756 |
| Chemical Hardness (η) | 3.7732 |
| Chemical Potential (μ) | 4.0488 |
| Electronegativity (χ) | -4.0488 |
| Softness (*S*) | 0.2650 |
| Electrophilicity Index (ω) | 2.1722 |

**Table S11: Physicochemical property analysis**

| **property** | **Octanoic acid** |
| --- | --- |
| Mol. Wt. (g/mol) | 144.21 |
| TPSA | 37.30 Å |
| ROB | 6 |
| HBD | 1 |
| HBA | 2 |
| Log P_o/w_ (iLOGP) | 1.95 |
| Log P_o/w_ (MLOGP) | 1.96 |
| Log S (ESOL) | -2.26 |
| Druglikeness  (Lipinski rule of five) | Yes; 0 violation |

**Table S12: Features and findings compared to previous studies**

| **Feature** | **Nair et al., 2005** | **Lin et al., 2023** | **Balta et al., 2024** | **Current Study** |
| --- | --- | --- | --- | --- |
| Antimicrobial Agent | Caprylic Acid (OA) & Monocaprylin | Octanoic Acid (OA) | AuraShield (Natural Antimicrobial Mixture) | Octanoic Acid (OA) |
| Primary Research Focus | Initial Efficacy: Inactivation of major mastitis pathogens in milk. | Biofilm/Persister Eradication: Clearance of antibiotic-tolerant cells and mature *S. aureus* biofilms. | Host Adherence/Inflammation: Mechanistic role in preventing *S. aureus* invasion/adherence in MAC-T cells. | Multimodal Molecular Mechanisms (Global Transcriptomics, Membrane damage, Host AMP regulation, and Computational Modeling). |
| Core Finding | Rapid Bactericidal against 5 mastitis pathogens (*S. aureus, E. coli, Strep* spp.) resulting in 5-8 log reduction in 1 hour. | Eradication of Biofilms and rapid killing of persistent biofilm cells and antibiotic-tolerant cells. | Anti-Adherence/Anti-Inflammatory effect at sub-inhibitory concentration; reduces bacterial invasion and pro-inflammatory cytokines (IL-6, IL-1B). | Global transcriptome profiling to find out the mechanism of action of OA. Comprehensive Mechanism: Rapid killing, Toxin Neutralization, Host Defense Upregulation, and anti-adherence |
| Mechanism of Action Analysis | None (Focus purely on efficacy/killing rate). | Targeted gene expression (qPCR) of key virulence regulators (*hla, agrA, sarA*). | Targeted gene expression (qPCR) of adhesion factors (ClfB); Anti-apoptotic assays. | RNA-seq analysis of *S. aureus* detailing hundreds of differentially expressed genes. Molecular docking, Dynamics (MD) simulations and Quantum Chemical (DFT) studies validate membrane disruption and compound stability |
| Anti-Virulence & Host Findings | None reported. | Inhibits biofilm formation; attenuates tetracycline-induced virulence factor gene expression (*hla*). | Blocks ClfB-Annexin A2 interaction to prevent adherence; preserves host cell viability (anti-apoptotic). | Mitigating toxicity and Inhibition of Toxin Assembly. Upregulates multiple Host Antimicrobial Peptide (AMP) genes (TAP, LAP, DEFB1, BNBD-4) in host cells |
| Resistance Studies | None reported. | Low propensity to induce Small Colony Variants (SCVs) or resistance after treatment. | Not explicitly tested. | Extensive study showing low propensity to induce resistance even after 30 passages |


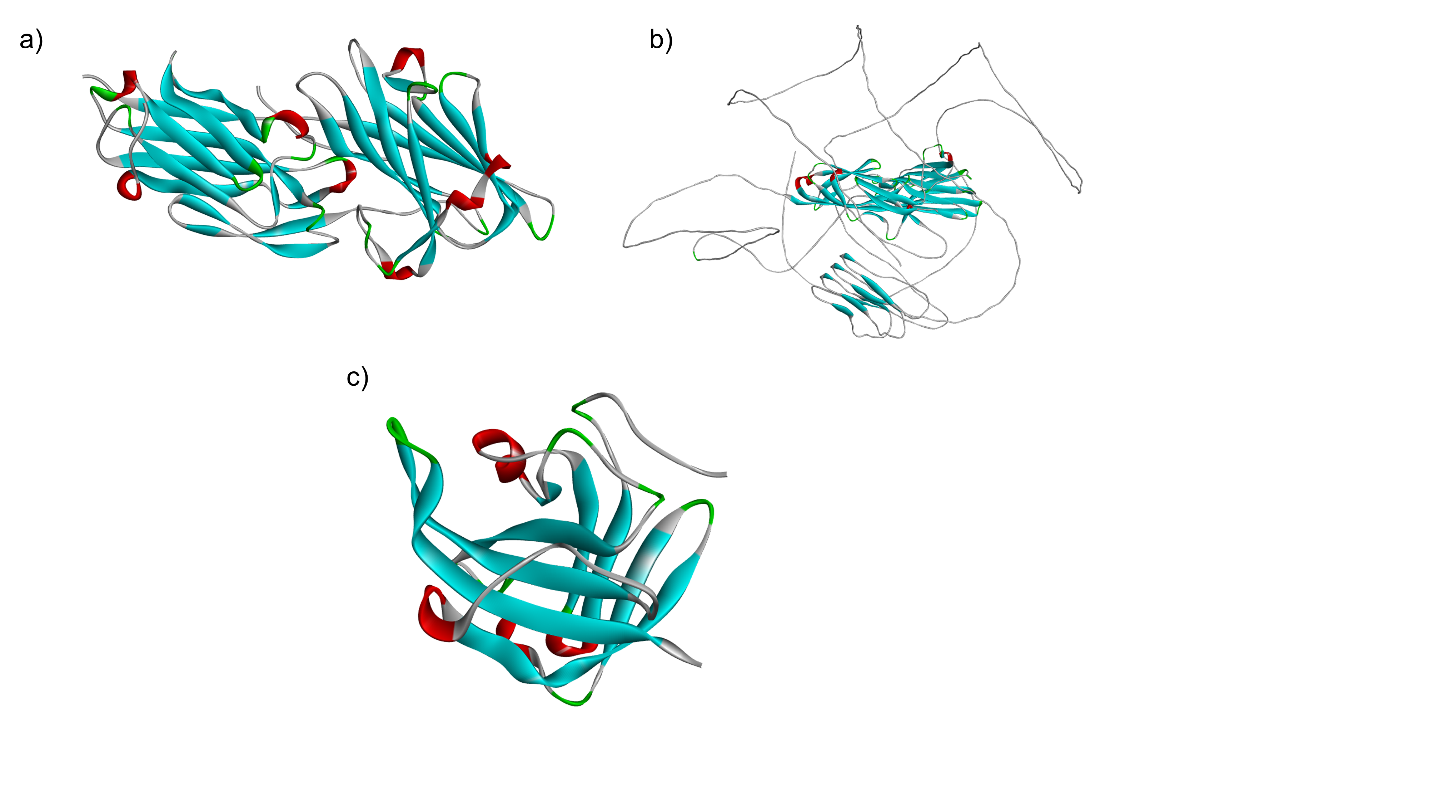


**Figure S1. 3D structures of the *S. aureus* adhesion proteins used in this study.** a) clumping factor B (PDB id: 4F20), b) fibronectin binding protein (FnBP) (ID: AF-A0A0H2XKG3), and c) sortase (PDB id: 1T2P).

**
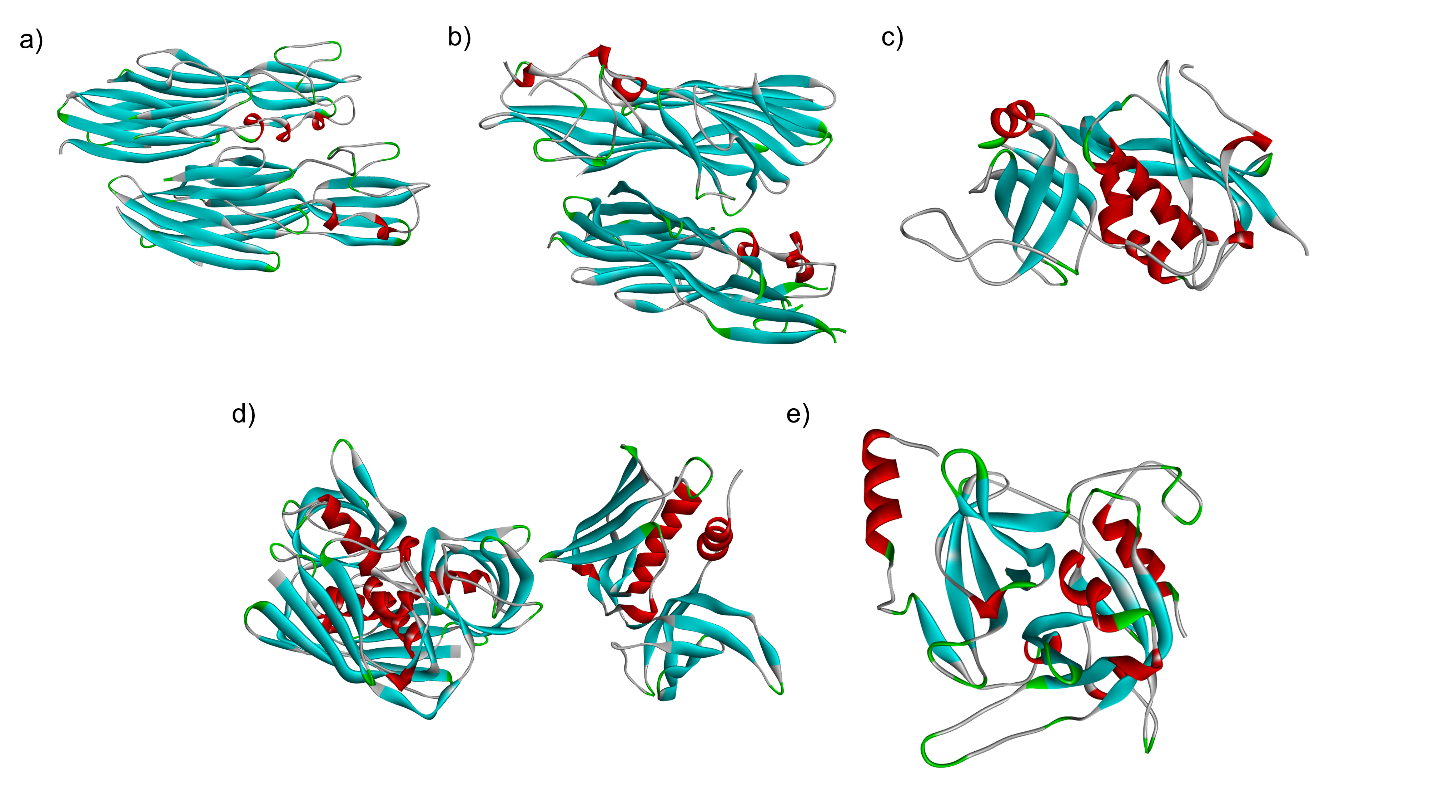
**

**Figure S2. 3D structures of the *S. aureus* toxins used in this study.** a) α-hemolysin monomer (PDB ID: 6U3T), b) γ-hemolysin monomer containing S and F subunits (PDB ID: 2QK7), c) enterotoxin B (PDB ID: 1SBB), d) toxic shock syndrome toxin (TSST) (PDB ID: 2QIL), and e) exfoliate toxin B (PDB ID: 1QTF).

**Figure S3: Effect of OA on cell viability of Mac-T cells.**

**
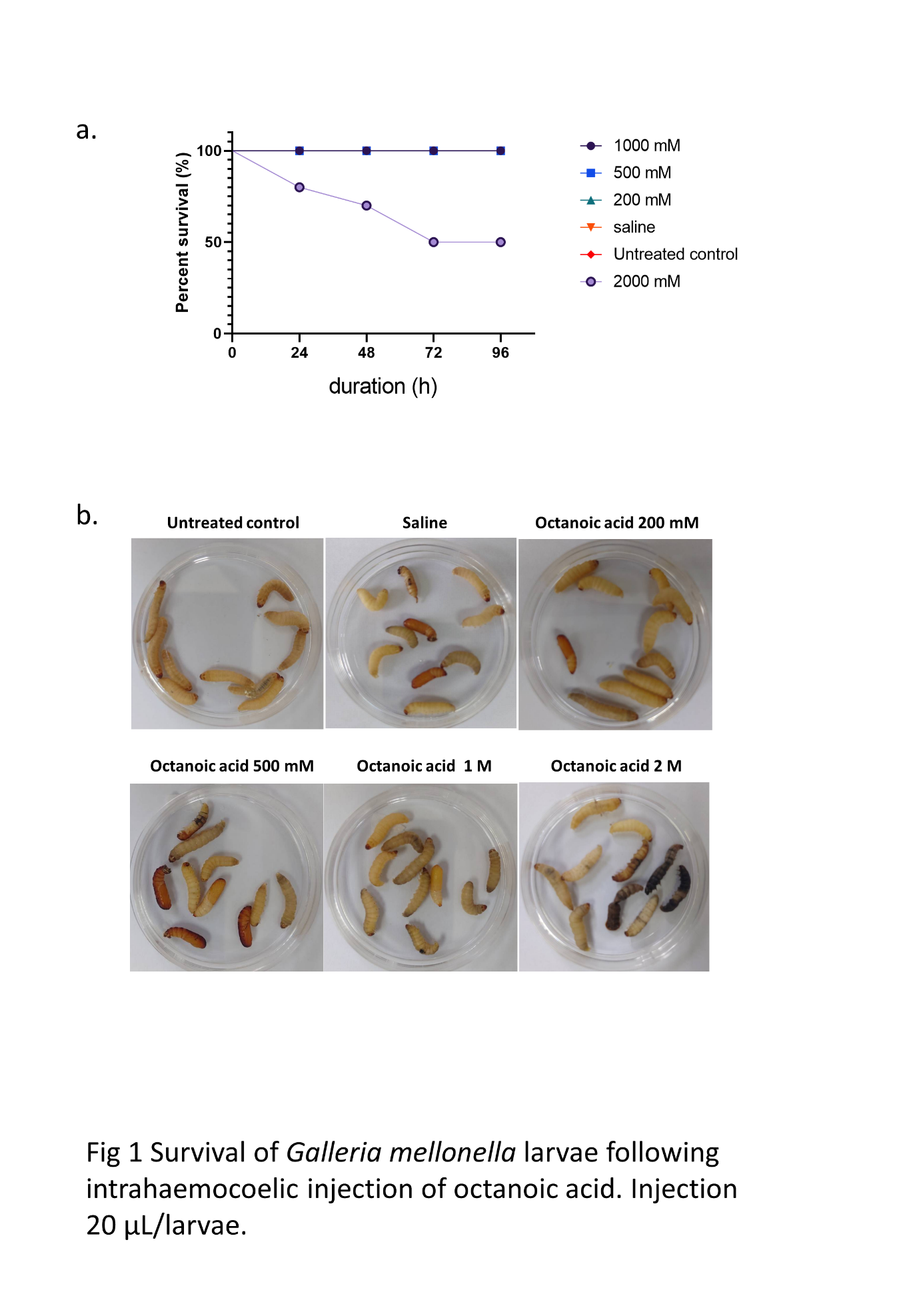
**

**Fig S4: Survival of *Galleria mellonella* larvae following intrahaemocoelic injection of octanoic acid.** Injection 20 μL/larvae. a) Survival g raph; b) worm observation after 96h


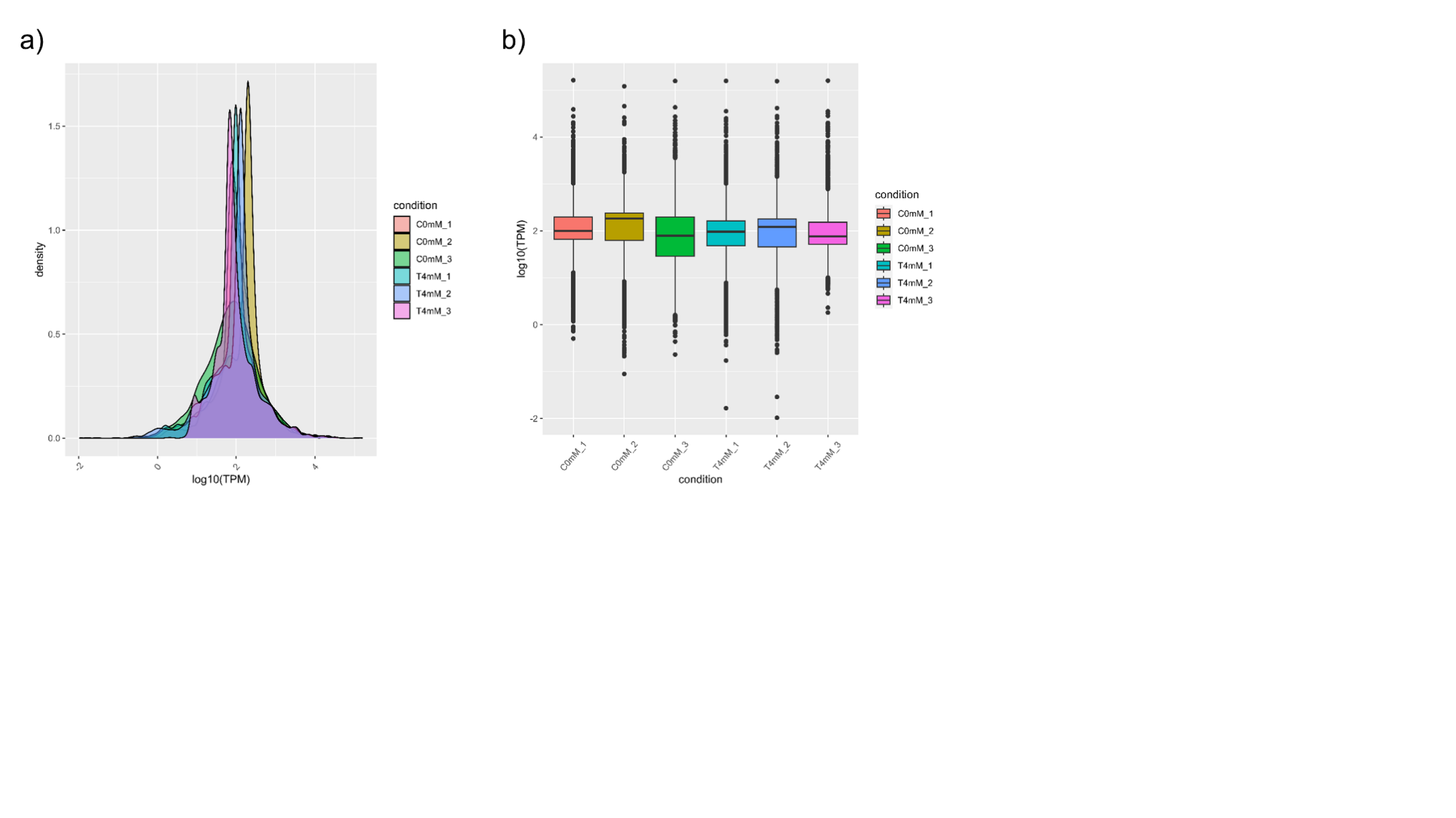


**Figure S5.** **Transcripts per million (TPM) analysis**. a) density distribution of genes; b) TPM box plots for control and OA treated cells


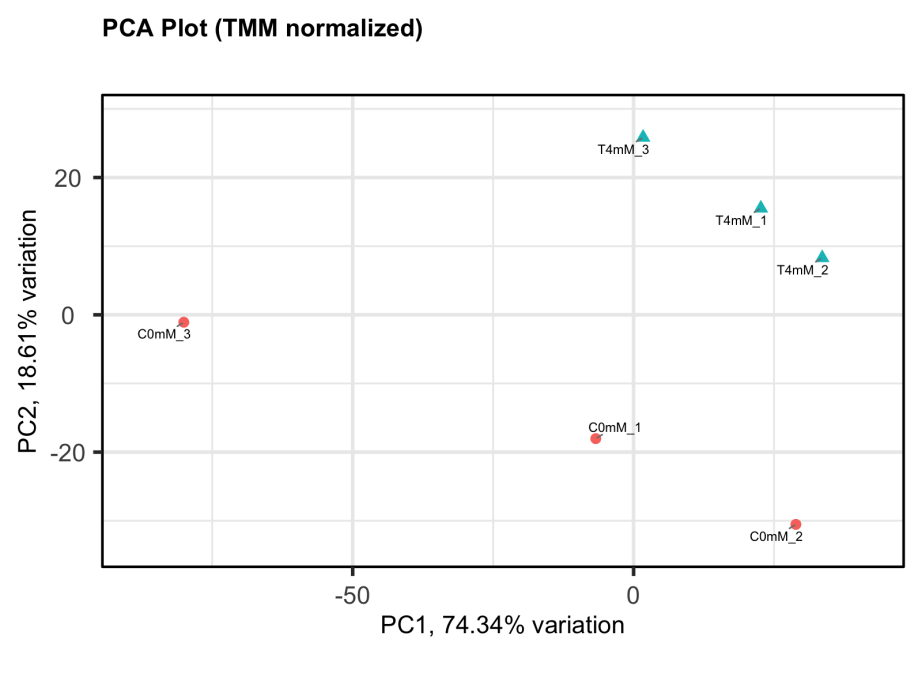


**Figure S6. Principal component analysis of the sample groups.**

**
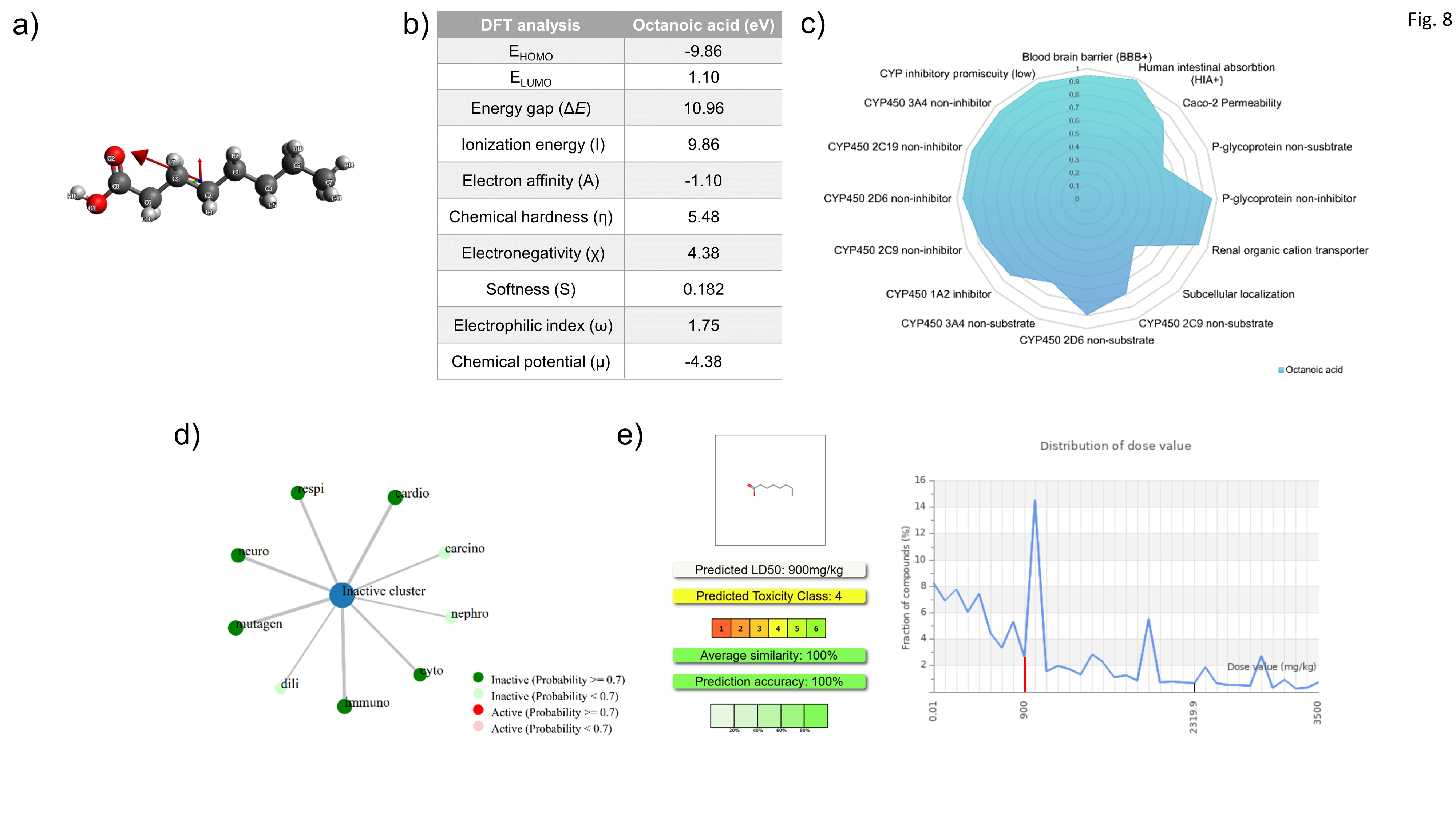
**

**Figure S7. Optimized structure of octanoic acid (OA)**
